# Transient upregulation of IRF1 during exit from naive pluripotency confers viral protection

**DOI:** 10.15252/embr.202255375

**Published:** 2022-07-19

**Authors:** Merrit Romeike, Stephanie Spach, Marie Huber, Songjie Feng, Gintautas Vainorius, Ulrich Elling, Gjis A Versteeg, Christa Buecker

**Affiliations:** ^1^ Max Perutz Labs Vienna Vienna Biocenter (VBC), University of Vienna Vienna Austria; ^2^ Vienna Biocenter PhD Program A Doctoral School of the University of Vienna and Medical University of Vienna Vienna Austria; ^3^ Institute of Molecular Biotechnology of the Austrian Academy of Science (IMBA) Vienna Biocenter (VBC) Vienna Austria

**Keywords:** pluripotency, viral infection, cell fate transition, ISG, gene regulatory networks, Microbiology, Virology & Host Pathogen Interaction, Signal Transduction, Stem Cells & Regenerative Medicine

## Abstract

Stem cells intrinsically express a subset of genes which are normally associated with interferon stimulation and the innate immune response. However, the expression of these interferon‐stimulated genes (ISG) in stem cells is independent from external stimuli such as viral infection. Here, we show that the interferon regulatory factor 1, *Irf1*, is directly controlled by the murine formative pluripotency gene regulatory network and transiently upregulated during the transition from naive to formative pluripotency. IRF1 binds to regulatory regions of a conserved set of ISGs and is required for their faithful expression upon exit from naive pluripotency. We show that in the absence of IRF1, cells exiting the naive pluripotent stem cell state are more susceptible to viral infection. *Irf1* therefore acts as a link between the formative pluripotency network, regulation of innate immunity genes, and defense against viral infections during formative pluripotency.

## Graphical abstract

Upregulation of the interferon regulatory factor 1 (Irf1) during the exit from naïve pluripotency is directly regulated by the cell state‐specific gene regulatory pluripotency network. Loss of Irf1 leads to increased susceptibility to viral infection.

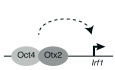

## Introduction

Mouse embryonic stem cells (ESCs) are self‐renewing in a naive pluripotent cell state *in vitro*. The essential gene regulatory network required to maintain naive pluripotency has been the subject of numerous studies and is very well‐defined (Betschinger *et al*, 2013; Dunn *et al*, 2014; Leeb *et al*, 2014). Development although is characterized by tightly regulated cell fate transitions, which are required to establish the remarkable complexity of multicellular organisms. After exiting naive pluripotency, cells enter a transient state termed formative pluripotency (Smith, 2017). This transition is characterized by global reorganization of the enhancer landscape and upregulation of the formative gene regulatory network (Buecker *et al*, 2014a). However, factors involved in this formative pluripotency gene regulatory network are less well‐understood: Genetic screens interrogating this transition have mostly focused on maintenance of (Li *et al*, 2018; Seruggia *et al*, 2019) or exit from naive pluripotency (Betschinger *et al*, 2013; Leeb *et al*, 2014; Li *et al*, 2018; Lackner *et al*, 2021). Formative pluripotency was only considered in the context of a multistep differentiation into primordial germ cell fate (Hackett *et al*, 2018).

Pluripotent and multipotent stem cells intrinsically express subsets of genes which are referred to as interferon‐stimulated genes (ISGs; Wu *et al*, 2018). The subsets of expressed ISGs are distinct between different stem cell types and change in differentiation (Wu *et al*, 2018). How these ISGs are regulated is unknown, as mouse ESCs for example do not respond to interferon (IFN) stimulation (Burke *et al*, 1978; Harada *et al*, 1990; Wang *et al*, 2013; Chen *et al*, 2020). Strikingly, pluripotent stem cells are more resistant against viral infection compared with their differentiated descendants (Swartzendruber & Lehman, 1975; Wolf & Goff, 2009), and this is at least in part due to ISG expression (Wu *et al*, 2018). However, whether ISGs and pluripotency gene expression are functionally connected has remained unknown.

Here, we identify the ISG *Irf1* in a CRISPR‐KO screen as a regulator of an enhancer that controls the expression of *Oct6*, a formative marker gene. IRF1 has a conserved role as regulator of other ISGs. Interestingly, many ISGs are differentially expressed between naive and formative pluripotency, and we show that IRF1 is directly responsible for the expression of a subset of these genes. Furthermore, IRF1 confers viral protection to cells during the exit from naive pluripotency. Finally, transient *Irf1* expression in formative pluripotent cells is directly regulated by the formative pluripotency gene regulatory network through a stem cell‐specific enhancer. Pluripotency and interferon responsive gene regulatory networks are therefore connected through *Irf1* during the transition from naive to formative pluripotency.

## Results

### Monitoring exit from naive and entry into formative pluripotency with a dual fluorescent reporter

We designed a fluorescent reporter system which simultaneously monitors exit from naive and entry into formative pluripotency. Murine ESCs are cultured under defined 2i + LIF conditions, which preserve the naive state of pluripotency. Upon withdrawal of 2i + LIF and stimulation with FGF2, the cells differentiate within 48 h irreversibly from the ESC state into epiblast‐like cells (EpiLC; Hayashi *et al*, 2011; Buecker *et al*, 2014a), often referred to as the formative state of pluripotency (Smith, 2017). Two differentially active enhancer elements (Appendix Fig S1A) were cloned into reporter constructs to activate different fluorescent markers. Thereby, we could follow the exit from naive pluripotency and the entry into formative pluripotency simultaneously: a naive‐specific enhancer close to *Tbx3* (*eTbx3*) controls GFP expression and a formative‐specific enhancer close to *Oct6* (also known as *Pou3f1*, *eOct6*) drives mCherry expression (Buecker *et al*, 2014a). Both constructs and *Cas9* cDNA were introduced into ESC, and two independent clonal cell lines were selected which showed clear separation of ESC and EpiLC populations after 48 h of differentiation by FACS analysis (Appendix Fig S1B–D). We tested the screening set‐up through deletion of *Tcf7l1* (also known as *Tcf3*): Loss of the transcription factor *Tcf7l1* causes a strong delay in the exit from naive pluripotency (Wray *et al*, 2011). As expected, *Tcf7l1* knockout (KO) caused a shift in dual fluorescent marker expression in the EpiLC population (Appendix Fig S1E), with mCherry showing lower expression than the WT cells and GFP showing higher expression in the *Tcf7l1* KO cells 48 h after initiation of differentiation. In summary, the dual fluorescent reporter enables faithful monitoring of the transition from naive into formative pluripotency.

### Screening for factors regulating entry into formative pluripotency

**Figure 1:**
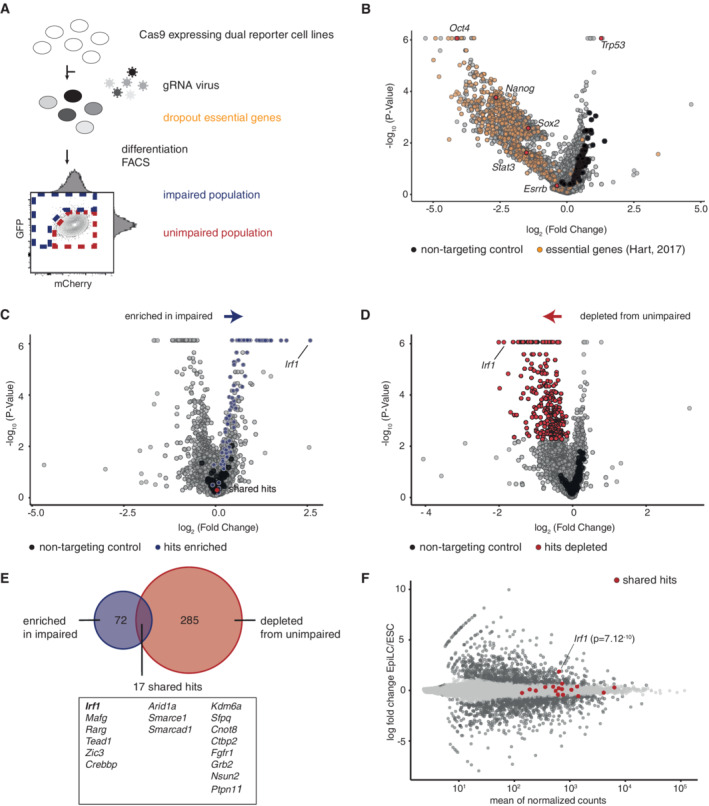
Screening for factors required for activation of formative pluripotency AOutline of CRISPR‐KO screening strategy. Reporter ESCs were transduced with a pooled CRISPR‐KO library. Cells selected for library integration with and without CAS9 were compared, assaying genes essential for the ESC state. Reporter cell lines were differentiated and scored by flow cytometry sorting as differentiation impaired (blue box) or unimpaired (red box). See also Fig EV1C and D for gating strategy.B–DVolcano plots of indicated comparisons in CRISPR‐KO screen analysis. The *x*‐axis represents fold change of sgRNA presence between indicated conditions, the *y*‐axis shows the binomial *P*‐value. Non‐targeting control sgRNA are indicated in black. All analyses are based on two replicates in two independent cell lines. (B) sgRNA representation in ESCs with CAS9 vs. ESCs without CAS9. Indicated are known ESC essential genes (Hart *et al*, 2017) and selected factors. See also Fig EV1B. (C) sgRNA representation in EpiLC sorted as impaired vs. non‐sorted EpiLC. Indicated in blue are factors enriched with FDR < 0.1. See also Fig EV1E. (D) sgRNA representation in EpiLC sorted as unimpaired vs. non‐sorted ESCs. Indicated in red are factors depleted with FDR < 0.1. See also Fig EV1F.EOverlap of all factors enriched in impaired and/or depleted from unimpaired populations, candidate factors found under both conditions are indicated.FMA plot of gene expression changes in EpiLC vs. ESC. The candidate factors which were identified as shared between both screening conditions (Fig 1E) are indicated in red. *P*‐value for *Irf1* calculated with DESeq2. *n* = 2 biological replicates.

**Figure EV1:**
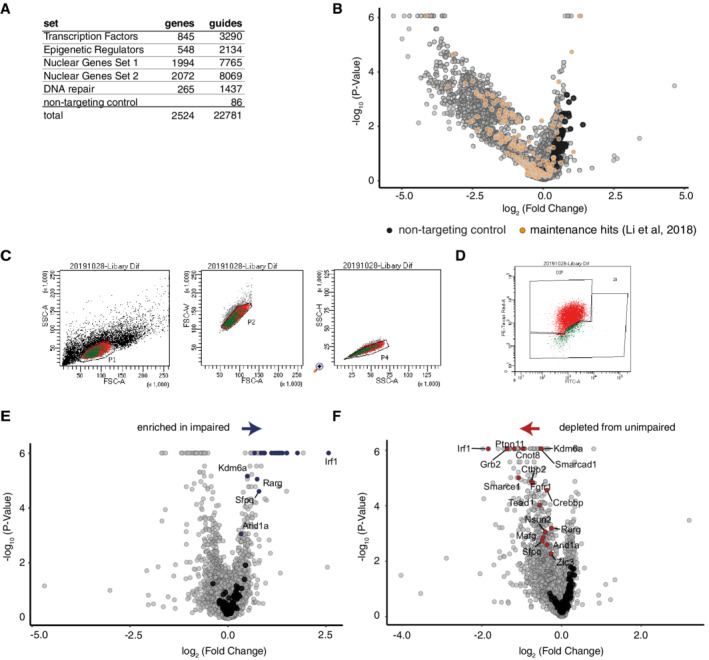
Screen strategy to identify factors required for activation of formative pluripotency AInformation about sgRNA library used in CRISPR‐KO screen. Shown are number of targeted genes and unique guides used for each class of genes.BAs in Fig 1B, sgRNA representation in ESCs with CAS9 vs. ESCs without CAS9. Indicated are known ESC maintenance genes (Li *et al*, 2018).CExample screening FACS gating strategy for CRISPR‐KO screen for selection of single cells. Live cells were sorted according to FSC‐A/SSC‐A gates, followed by gating for FSC‐A/FSC‐W and SSC‐A/SSC‐H for individual cells.DExample for gating strategy to sort impaired and unimpaired populations. Differentiation was scored by FITC‐A/PE‐TexasRed‐A gating.EAs Fig 1C, sgRNA representation in EpiLC sorted as impaired vs. non‐sorted EpiLC. Indicated in blue are factors identified as shared candidates (see Fig 1E).FAs Fig 1D, sgRNA representation in EpiLC sorted as unimpaired vs. non‐sorted ESCs. Indicated in red are factors identified as shared candidates (see Fig 1E).

Next, we performed a pooled CRISPR KO screen with the dual reporter cell line to identify factors regulating the entry into formative pluripotency. We transduced the reporter cell lines (100 million cells) with a lentiviral library for the expression of 22,781 unique sgRNAs targeting 2,524 nuclear localized genes, including 159 genes considered ISGs (Figs 1A and EV1A, Dataset EV1, Dataset EV3, Dataset EV4). We expected a candidate regulator of formative pluripotency to modify transcription and such a small, selected library increases statistical robustness. We also infected wild‐type ESC (R1 cells) lacking CAS9 expression to control for sgRNA abundance in the lentiviral library. We selected infected cells with Neomycin for 48 h. After recovery from selection for an additional 48 h, we harvested 60 million cells to identify ESC essential factors: We compared the abundance of sgRNA coding sequences in genomic DNA from CAS9 expressing or non‐expressing cells. As expected, we observed enrichment of *Trp53* sgRNA, as cells lacking *p53* proliferate faster and enrich in populations of mixed clones (Sabapathy, 1997; Fig 1B). Conversely, ESCs lacking core pluripotency factors including OCT4 (also known as POU5F1) and NANOG depleted from this population, as these factors are required for naive pluripotency (Fig 1B). Moreover, larger sets of known essential factors showed depletion (Figs 1B and EV1B; Hart *et al*, 2017; Li *et al*, 2018). We conclude that the overall screening set‐up could identify known essential regulators of the naive pluripotent stem cell state.

Next, we applied our screening platform to identify potential activators of the formative pluripotency network. We differentiated ESCs for 48 h into EpiLC and binary scored EpiLCs as differentiation “impaired” or “unimpaired”: Cells with reporter activity overlapping the behavior of no sgRNA controls were FACS‐sorted as unimpaired. Conversely, cells with higher *eTbx3* controlled GFP and/or lower *eOct6* controlled mCherry were sorted as impaired (see Figs 1A, and EV1C and D for gating strategy). In addition, we also collected unsorted EpiLCs as baseline. This strategy allowed several comparisons of sgRNA abundance: KO of a candidate driving the formative state should increase the probability of impaired differentiation; therefore, the sgRNAs should be enriched in the impaired populations. This was the case for a total of 72 factors (Fig 1C). Conversely, a candidate KO should be incompatible with unimpaired differentiation, and therefore, sgRNAs drop out from the unimpaired population. Two hundred and eighty‐five factors showed such a behavior (Fig 1D). We identified an overlapping set of 17 hits, which were found in both comparisons and are therefore the most stringent candidates involved in this cell fate transition (Figs 1E, and EV1E and F). Among these candidates were members of pathways with known involvement in stem cell differentiation: The FGF receptor *Fgfr1*, *Tead1* which plays a role in the Hippo pathway (Molotkov *et al*, 2017) and *Zic3*, a known regulator of the exit from naive pluripotency and entry into primed pluripotency (Yang *et al*, 2019).

In addition, we identified several members of the SWI/SNF chromatin remodeling family, also called BAF complexes (*Arid1a*, *Smarce1*, and *Smarcad1*). Specific subunits of BAF complexes are connected to diverse phenotypes in ESCs (reviewed in Ye *et al*, 2021). SMACRCE1/BAF57 is a canonical BAF subunit, and ARID1A/BAF250A is a component of the ESC‐specific esBAF, with *Arid1a*‐deficient mice showing an arrest in early embryonic development (Gao *et al*, 2008). SMARCAD1 has been shown to silence endogenous retrovirus in ESCs (Sachs *et al*, 2019). In sum, we were able to identify known regulators of the exit from naive pluripotency, demonstrating that our screening approach can identify factors involved in the exit from naive pluripotency.

One of the top screening hits was the interferon regulatory factor 1, *Irf1*, a transcription factor and a member of ISGs. *Irf1* was depleted from the unimpaired cell population and enriched in the impaired population for every single analyzed condition. We validated gene expression levels for all hits in ESC and EpiLC cells (Fig 1F). All hits are expressed, but only *Irf1* shows significant upregulation in differentiation. Therefore, we focused on *Irf1* and explored its function during the exit from naive pluripotency.

### *Irf1* is activated by the formative pluripotency gene regulatory network

**Figure 2:**
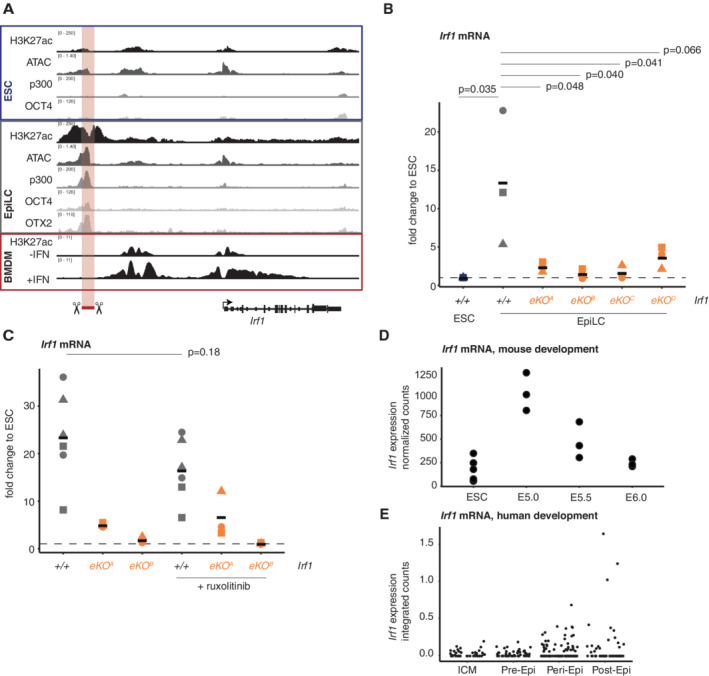
Formative pluripotency drives transient *Irf1* expression AChromatin context of *Irf1* in ESCs, EpiLC and BMDMs with and without IFNɣ stimulation. Shown are ChIP‐seq tracks of histone mark H3K27ac, transcription factors OCT4, OTX2, and p300 and ATAC seq profiles (data were generated by Data ref: Buecker *et al*, 2014b; BMDM Data ref: Langlais *et al*, 2016b). The putative enhancer that was knocked out is indicated.BRT‐qPCR analysis of *Irf1* mRNA in *Irf1* ^+/+^ and *Irf1* enhancer KO in differentiation. Fold change is normalized against *Rpl13a* housekeeping mRNA expression and is calculated against ESC for each indicated cell line. This is shown as baseline with the dashed line at fold change = 1. Statistical tests were performed as homoscedastic one‐sided *t*‐tests. Shown are three biological replicates, datapoints from the same replicate are indicated with the same symbol. Black horizontal lines show the mean of the data.CRT‐qPCR analysis of *Irf1* mRNA in *Irf1* ^+/+^ and *Irf1* enhancer KO in differentiation, treated with ruxolitinib. Fold change is normalized against *Rpl13a* housekeeping mRNA expression and is calculated against ESC for each indicated cell line. This is shown as baseline with the dashed line at fold change = 1. Statistical test was performed as homoscedastic one‐sided *t*‐tests. Shown are three biological replicates, datapoints from the same replicate are indicated with the same symbol. Note that each replicate contains two *Irf1* ^+/+^ samples. Black horizontal lines show the mean of the data.D
*Irf1* expression in murine blastocysts and ESCs (Data ref: Kinoshita *et al*, 2021b). For E5.0, E5.5 and E6.0, entire single blastocysts were sequenced (*n* = 3 biological replicates), for ESCs *n* = 4 biological replicates.E
*Irf1* expression in human blastocysts. Integrated single‐cell RNA‐seq data from blastocysts collected at or cultured to indicated time points. Data are derived from a total of 16 embryos which passed quality control metrics (Data ref: Molè *et al*, 2021b).

As *Irf1* is upregulated during exit from naive pluripotency, we analyzed the chromatin environment surrounding the *Irf1* gene to identify potential regulatory regions that might control *Irf1* expression in EpiLCs. We identified a putative enhancer region 10 kb upstream of *Irf1*. In ESCs, this element is not strongly marked by active enhancer marks. However, in EpiLCs, this element is marked by OCT4, P300, the formative transcription factor OTX2 and the active histone modifications H3K27ac and H3K4me1 (Fig 2A). KO of *Otx2* has limited effect on the exit from naive pluripotency, but *Otx2* KO EpiLCs have significantly lower expression of *Irf1*, suggesting that the pluripotency network controls the expression of *Irf1* (Buecker *et al*, 2014a). Importantly, this putative enhancer is specific for the ESC to EpiLC transition as it was not marked by H3K27ac in unstimulated and IFN‐ɣ exposed bone marrow‐derived macrophages (BMDMs). In contrast, a region 6 kb upstream of *Irf1* is marked by increasing H3K27ac in BMDMs after IFN‐ɣ stimulation. This suggests a cell type‐specific regulation mechanism for *Irf1* during the exit from naive pluripotency.

**Figure EV2:**
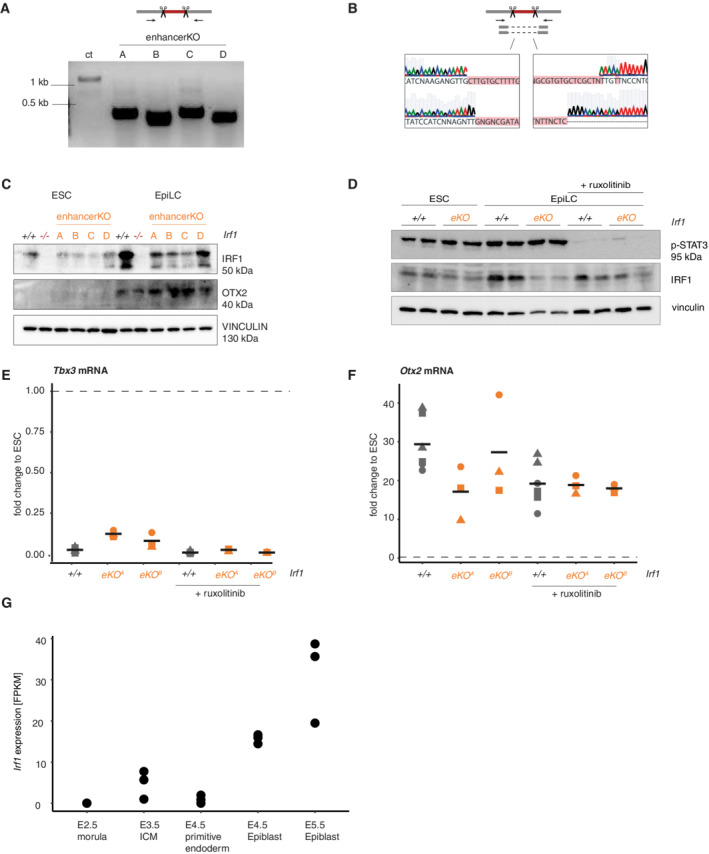
IRF1 expression in formative pluripotency is controlled by an EpiLC‐specific enhancer AGenotyping PCR of enhancer KO. Primers bind outside the edited site, producing smaller PCR products than on the control locus. Enhancer KO A and C were generated with one set of sgRNA, enhancer KO B and D with a second, different set.BExamples for Sanger sequencing of PCR products, shown are enhancer KO B and D. Gapped alignment is indicated by dashed lines.CWestern blot analysis of *Irf1* ^+/+^, I*rf1* ^−/−^ and *Irf1* enhancer KO ESC and EpiLC, probed with antibodies against IRF1, formative marker OTX2 and VINCULIN as loading control.DWestern blot analysis of *Irf1* ^+/+^ and *Irf1* enhancer KO ESC and EpiLC with ruxolitinib treatment, probed with antibodies against phosphorylated STAT3, IRF1 and VINCULIN as loading control.E, FRT‐qPCR analysis of *Tbx3* (E, naive marker) and *Otx2* (F, formative marker) mRNA in *Irf1* ^+/+^ and *Irf1* enhancer KO in differentiation, treated with ruxolitinib. Fold change is normalized against *Rpl13a* housekeeping mRNA expression and is calculated against ESC for each indicated cell line. This is shown as baseline with the dashed line at fold change = 1. Shown are three biological replicates, datapoints from the same replicate are indicated with the same symbol. Note that each replicate contains two *Irf1* ^+/+^ samples. Black horizontal lines show the mean of the data.G
*Irf1* expression [FPKM] in murine embryos (Data ref: Boroviak *et al*, 2015b); *n* = 3 biological replicates.

We deleted the identified putative enhancer region to analyze whether the element indeed controls the expression of *Irf1*. We generated four independent enhancer KO cell lines and validated the absence of the enhancer region by genotyping PCRs and sequencing of the PCR products (Fig EV2A and B). We then tested whether *Irf1* expression is perturbed during differentiation into EpiLCs upon enhancer loss. Indeed, enhancer KO lines showed significantly reduced *Irf1* mRNA levels (Fig 2B). We also confirmed reductions in IRF1 protein levels by Western blot (Fig EV2C). The formative marker OTX2 is not influenced by the enhancer KO, and lack of IRF1 expression is therefore not caused by lack of transition to the EpiLC state. We conclude that *Irf1* expression in EpiLCs is directly connected to the formative network by enhancer regulation.

In the IFN response, JAK/STAT signaling is a known regulator of *Irf1* expression and JAK/STAT is also active in naive and formative pluripotency. We therefore tested whether JAK/STAT signaling activates *Irf1* expression in formative pluripotency. We treated cells with the JAK inhibitor ruxolitinib at the onset of differentiation. Treatment drastically reduced phosphorylation levels of STAT3 (Fig EV2D) but did not interfere with differentiation, as *Tbx3* and *Otx2* as naive and formative markers, respectively, were not misregulated (Fig EV2E and F). *Irf1* mRNA levels were not significantly reduced by ruxolitinib treatment in WT cells (Fig 2C), and ruxolitinib did not decrease *Irf1* expression further in enhancer KOs (Fig 2C). Together, these results demonstrate that direct action of the formative pluripotency network, and not IFN signaling, is the main driver of *Irf1* expression during the exit from naive pluripotency.

### Transient IRF1 expression in the epiblast is conserved

We wondered whether upregulation of *Irf1* during the exit from naive pluripotency is limited to the *in vitro* 2D cell culture differentiation system. Therefore, we analyzed publicly available RNA‐seq data collected from peri‐ and early postimplantation murine embryos at days E5.0, E5.5, and E6.5 (Fig 2D; Kinoshita *et al*, 2021a; Data ref: Kinoshita *et al*, 2021b). *Irf1* expression is transiently increased during implantation at E5.0, but rapidly decreases again within 1 day of development. *Irf1* is specific to the epiblast, as E4.5 primitive endoderm cells are not showing *Irf1* expression (Fig EV2G; Boroviak *et al*, 2015a; Data ref: Boroviak *et al*, 2015b). Accordingly, *Irf1* expression in a single cell dataset spanning mouse gastrulation is highest in epiblast and primitive streak metacells and downregulated in later states (Mittnenzweig *et al*, 2021). We conclude that the expression of *Irf1* is transiently upregulated in implantation stage embryos; therefore, the expression of *Irf1* in EpiLCs reflects the *in vivo* expression patterns observed in murine peri‐implantation epiblasts.

We further asked whether *Irf1* expression is conserved in humans. Due to the limited availability of human embryonic data, we used a published dataset which integrates preimplantation single‐cell expression data with data collected from blastocysts derived at 5 d.p.f. and cultured until 9 and 11 d.p.f., representing the pre‐ and postimplantation epiblast (Molè *et al*, 2021a; Data ref: Molè *et al*, 2021b). Cells showed an increase in *Irf1* expression in peri‐ and early postimplantation epiblasts (Fig 2E). The transient expression of *Irf1* is a conserved feature of implantation between murine and human embryogenesis.

### IRF1 is a regulator of interferon‐stimulated genes during pluripotency

**Figure 3:**
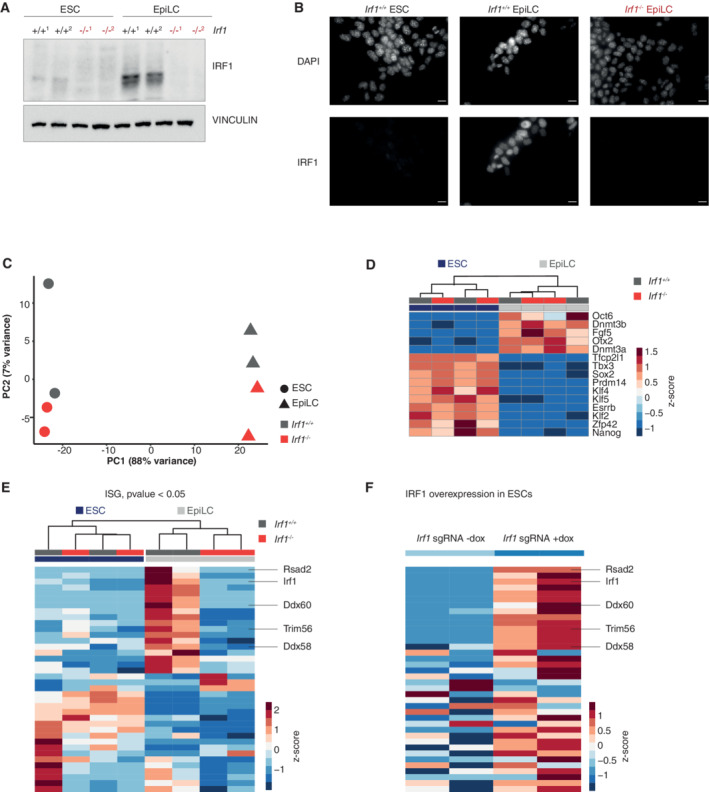
IRF1 regulates ISG expression in EpiLCs AWestern blot analysis of *Irf1* ^−/−^ in ESC and EpiLC, probed with antibodies against IRF1 and VINCULIN as loading control. Shown are two independent clonal cell lines. See Fig EV3A for long exposure and size marker of the same blot.BImmunofluorescence with IRF1 antibody in *Irf1* ^+/+^ and *Irf1* ^−/−^ ESC and EpiLC. Nuclei are stained with DAPI. Scale bars are 10 μm.CDimension reduction (principal component analysis, PCA) of *Irf1* ^+/+^ and *Irf1* ^−/−^ ESC and EpiLC, based on QuantSeq RNA data. PCA captures global transcriptional states of samples and similarity of these states is reflected in closeness of samples in the plot. *n* = 2 biological replicates.DExpression changes in selected pluripotency markers in *Irf1* ^+/+^ and *Irf1* ^−/−^ ESC and EpiLC Quantseq RNA data. Data are shown as gene normalized *z*‐score. *n* = 2 biological replicates.EExpression changes of ISGs in *Irf1* ^+/+^ and I*rf1* ^−/−^ ESC and EpiLC Quantseq RNA data. Shown are ISGs differentially expressed (*P*‐value < 0.05) between *Irf1* ^+/+^ and *Irf1* ^−/−^ EpiLCs. Data are shown as gene normalized *z*‐score. See also Fig EV3E. Selected gene examples are indicated. *n* = 2 biological replicates.FExpression changes in the same genes as in Fig 3E in dox‐inducible SunTag‐based IRF1 overexpression. Data are shown gene normalized as *z*‐score. Selected gene examples are indicated. *n* = 2 biological replicates.

**Figure EV3:**
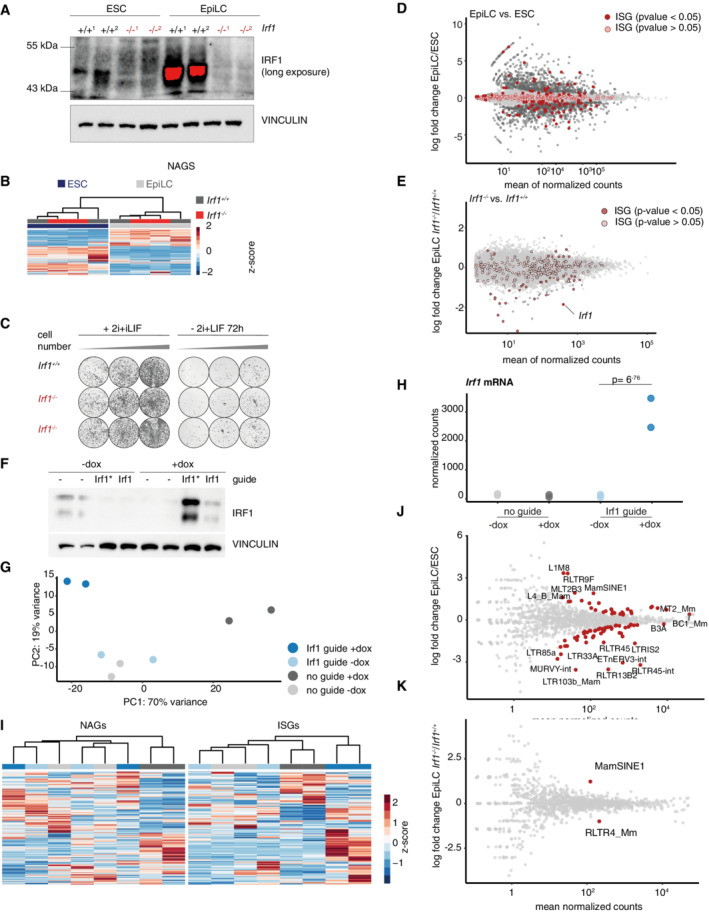
IRF1 is not a major regulator of pluripotency gene or repeat element expression ASame Western blot as Fig 3A, long exposure and size marker of IRF1, VINCULIN is used as loading control. Overexposure is indicated with red signal.BExpression changes in naive‐associated genes (NAGs, Lackner *et al*, 2021) in *Irf1* ^+/+^ and *Irf1* ^−/−^ ESC and EpiLC based on QuantSeq RNA data. Data are shown as *z*‐score. *n* = 2 biological replicates.CAlkaline phosphatase staining of *Irf1* ^+/+^ and *Irf1* ^−/−^ in different cell seeding densities. Left panel: Cells were kept in 2i + LIF medium to control for seeding density, right panel: Cells were differentiated for 72 h before being placed back in 2i + LIF medium.DMAplot of gene expression changes in EpiLC vs. ESC, ISGs are indicated. *n* = 2 biological replicates.EMAplot of gene expression changes in EpiLC *Irf1* ^−/−^ vs. *Irf1* ^+/+^, ISGs are indicated. Differentially expressed (*P*‐value < 0.05) Differentially expressed ISGs are plotted in Fig 3E. *n* = 2 biological replicates.FWestern blot analysis of dox‐inducible SunTag‐based IRF1 overexpression in ESCs, probed with antibodies against IRF1 and VINCULIN as loading control. Two different sgRNAs were tested, the sgRNA marked with an asterix (Irf1*) was used for RNA‐sequencing.GDimension reduction (principal component analysis, PCA) plot of dox‐inducible SunTag‐based IRF1 overexpression in ESCs, based on QuantSeq RNA data. *n* = 2 biological replicates.HNormalized counts for *Irf1* mRNA in dox‐inducible SunTag‐based IRF1 overexpression in ESCs, based on QuantSeq RNA data. *P*‐value calculated by DESeq2. *n* = 2 biological replicates.IExpression changes in dox‐inducible SunTag‐based IRF1 overexpression, left panel NAGS, right panel full set of ISGs. Data are shown as gene normalized *z*‐score. Samples are color‐coded as in Fig EV3G. *n* = 2 biological replicates.JMAplot of TE family expression changes in EpiLC vs. ESC, adjusted *P*‐values < 0.1 are indicated. *n* = 2 biological replicates.KMAplot of TE family expression changes in EpiLC *Irf1* ^−/−^ vs. *Irf1* ^+/+^, adjusted *P*‐values < 0.1 are indicated. *n* = 2 biological replicates.

Next, we wanted to understand the function of IRF1 in the exit from naive pluripotency and generated *Irf1* KO cell lines. We simultaneously transfected two CRISPR sgRNAs to delete an exon–intron boundary around intron 3 of *Irf1* into our dual reporter cell lines and R1 WT cells using lipofection. We identified individual clones through genotyping PCR and validated the absence of full‐length protein by Western blot (Figs 3A and EV3A). Control ESCs showed very low IRF1 levels, but in *Irf1* KO cells, the protein is absent. IRF1 is localized to the nucleus, with homogenous faint expression in ESCs and robust homogenous signals in EpiLCs (Fig 3B). This signal in EpiLCs is lost upon deletion of *Irf1* (Fig 3B).

Next, we asked whether IRF1 affects gene expression in differentiation from ESC to EpiLCs. We performed RNA‐seq using the Quantseq protocol for WT and two independent *Irf1* KO cell lines under ESC and EpiLC conditions (Dataset EV5). Principal component analysis captured differentiation along PC1 and genotype‐specific effects along PC2 (Fig 3C). These results indicate that *Irf1* KO cells do not show drastically changed cell states in differentiation. To corroborate these findings, we focused on well‐established pluripotency markers, expressed either in ESCs (such as *Klf4*, *Esrrb*, and *Nanog*), or in EpiLCs (for example, *Fgf5* and *Otx2*) and found little change in expression. With the exception of *Oct6*, which was reduced (Fig 3D; all shown factors *P*‐value control vs. *Irf1* KO > 0.05, besides Tbx3 (*P*‐value = 0.031)). Previously, a list of 496 genes which are either positively or negatively associated with the core pluripotency markers was described (naive‐associated genes, NAGS (Lackner *et al*, 2021)). NAGs are also correlated with pre‐ to postimplantation expression changes in mice and macaque *in vivo*. As expected, NAGs show strong ESC‐ and EpiLC‐specific expressions, but no drastic change upon *Irf1* deletion (Fig EV3B).

We tested whether *Irf1* KO cells displayed a general exit from naive pluripotency defect using alkaline phosphatase staining (Fig EV3C). We differentiated cells for 72 h to irreversibly exit naive pluripotency and plated them back under naive 2i + LIF conditions. Genotypes with exit from naive pluripotency defect show an increased fraction of cells still able to proliferate under 2i + LIF conditions (Leeb *et al*, 2014). *Irf1* KO cells did not show increased colony formation and therefore are not impaired in the exit from naive pluripotency. We conclude that IRF1 does not regulate exit from naive or entry into formative pluripotency.

*Irf1* is a member of a group of genes previously classified as ISGs. A subset of ISGs are intrinsically expressed in stem cells of several species, even in the absence of viral stimulation (Wu *et al*, 2018). In differentiation to somatic cell types, this intrinsic ISG expression and thereby viral protection is lost (Wu *et al*, 2018).

Given the role of IRF1 as a transcription factor and as a core component of the type I and II IFN responses, we asked whether ISG expression is influenced by IRF1 during the exit from naive pluripotency. We first confirmed high expression levels of ISGs (as defined in Wu *et al*, 2018) in our ESC and EpiLC RNA‐seq data (Fig EV3D). As expected, a subset of ISGs was expressed in both ESCs and EpiLCs; however, we also observed changes in the cell state‐specific expression of ISGs (Fig EV3D). Notably, *Irf7*, which was shown to be incompatible with maintenance of pluripotency, was absent in either cell state (Eggenberger *et al*, 2019). Next, we analyzed which ISGs are differentially expressed in *Irf1* KO vs. WT in EpiLCs (Fig EV3E). Out of the subset of differentially expressed ISGs, *Irf1* KO most strikingly affects genes which under normal differentiation conditions are upregulated (Fig 3E). Examples for ISGs which are not upregulated in *Irf*1 KO include *Ddx58*, *Ddx60*, *Rsad*2, and *Trim56* (see also below).

We asked whether changes in gene expression are directly controlled by IRF1 and established an IRF1 overexpression system in ESCs using an inducible CRISPR‐ON system (Heurtier *et al*, 2019). CRISPR sgRNAs are constitutively expressed. Upon doxycycline (dox) addition, dCAS9 coupled to 10× GCN4 is induced, which in turn recruits several molecules of scFv‐linked VP64, a potent transcriptional activator. We used an sgRNA targeting the *Irf1* promoter and validated the expression of full‐length IRF1 protein 48 h after dox induction in ESC (Fig EV3F). We performed QuantSeq to detect RNA expression changes after IRF1 overexpression (Dataset EV7). As a control, we included dox induction of dCAS9 in the absence of sgRNA expression. Activity of untargeted dCAS9 had a strong effect on gene expression, potentially because of DNA damage induction and off‐target effects (Fig EV3G; Tycko *et al*, 2019). As expected, IRF1 expression was upregulated when sgRNA directed to the promoter of *Irf1* was used under dox induction conditions (Fig EV3H). The expression of NAGs was not influenced by IRF1 overexpression, but rather by non‐targeted dCas9 presence (Fig EV3I, left panel). In contrast, ISG and specifically those ISG that were also misregulated in *Irf1* KO, showed upregulation in IRF1 overexpression (Fig EV3I, right panel, Fig 3F).

Pluripotency is associated with lack of epigenetic repression and transcriptional activation of transposable elements (TEs; Peaston *et al*, 2004). Transposable elements include endogenous retroviruses, and IRF1 is involved in repression of endogenous retroviruses (Stoltz *et al*, 2019). We analyzed expression levels of TE families in WT differentiation and in the absence of IRF1 in EpiLCs (Dataset EV6). ESCs and EpiLCs have distinct transcriptional profiles for TE families (Fig EV3J). In contrast, deletion of *Irf1* only has a minor effect on TE families (Fig EV3K). Therefore, we conclude that IRF1 is not a major regulator of TE expression in naive and formative pluripotency. However, *Irf1* is involved in the activation of a subset of ISGs.

### The Oct6 enhancer is directly regulated by IRF1

**Figure EV4:**
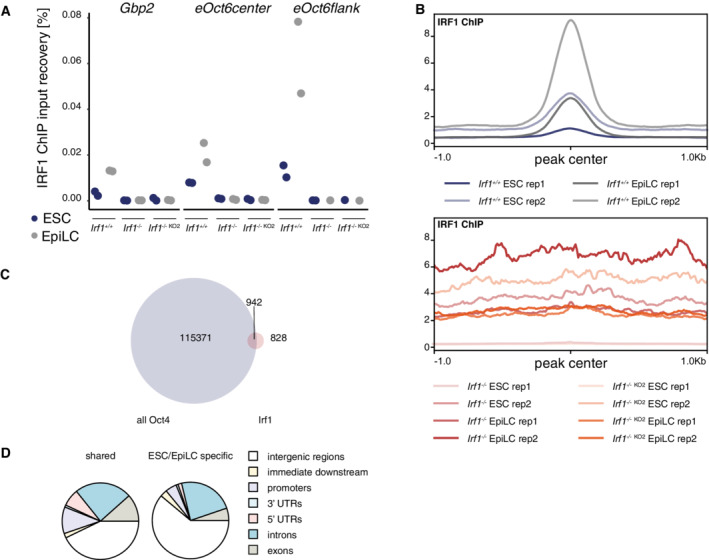
Chromatin binding of IRF1 AChIP‐qPCR analysis for the *Gbp2* promoter as known IRF1 binding site and two primer sets for the *eOct6* enhancer. Values are calculated as input recovery [%]. *n* = 2 biological replicates.BSignal strength around all identified IRF1 binding sites for indicated conditions. Top panel: *Irf1* ^+/+^ ESC and EpiLCs, bottom panel: two independent *Irf1* ^−/−^ cell lines in ESC and EpiLC conditions. *n* = 2 biological replicates.COverlap of IRF1 binding sites and all OCT4 binding sites in ESCs and EpiLCs. (Data ref: Buecker *et al*, 2014b).DAssigned chromatin regions of IRF1 binding sites shared between BMDMs and ESC/EpiLC (left) and IRF1 binding sites only detected in ESC/EpiLC (right).

Next, we asked which chromatin sites are bound by endogenous IRF1 in ESCs and EpiLCs. First, we validated IRF1 ChIP efficiency by qPCR. IRF1 ChIP recovers the promoter of *Gbp2* (Fig EV4A), a known IRF1 bound site in macrophages (Ramsauer *et al*, 2007; Langlais *et al*, 2016a). In addition, we also assayed two primer pairs at the *eOct6* enhancer locus, both showed enrichment in IRF1 Chip‐qPCR (Fig EV4A).

**Figure 4:**
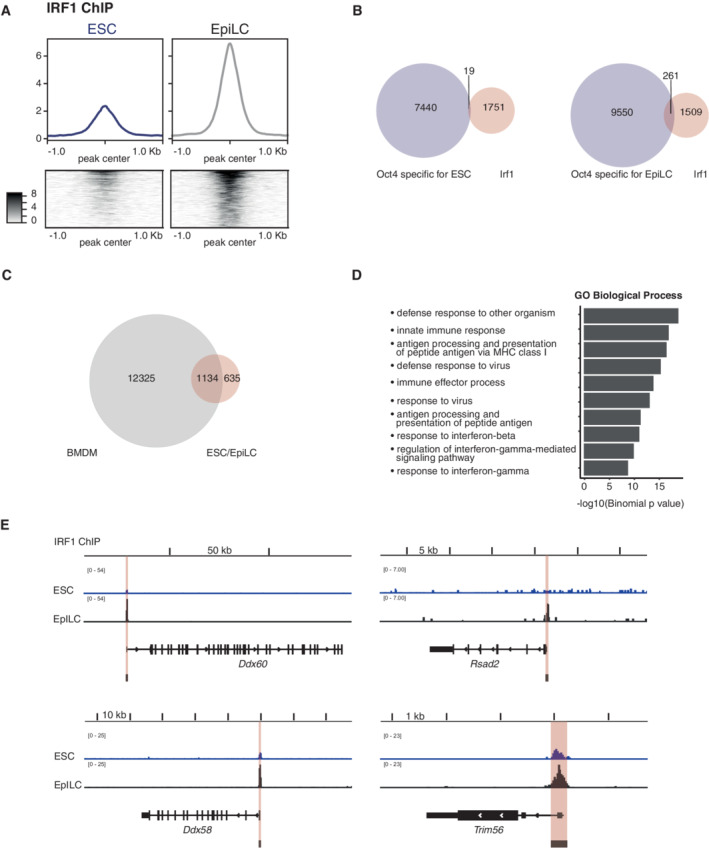
IRF1 binds to genes involved in the innate immune response AIRF1 ChIP‐seq in ESC and EpiLC. Binding profiles and heatmaps of the consensus of all binding sites are shown.BVenn diagram of overlap between chromatin IRF1 binding sites and OCT4 binding sites specific in ESCs (left) or EpiLCs (right). (Data ref: Buecker *et al*, 2014b).CVenn diagram of overlap between chromatin IRF1 binding sites identified in bone marrow‐derived macrophages (BMDM) −IFNɣ/+IFNɣ (Data ref: Langlais *et al*, 2016b) and ESC/EpiLC.DGREAT‐analysis of GO term enrichment for IRF1 chromatin binding sites in ESC/EpiLC. Binomial *P*‐values of top 10 GO biological processes are shown.EChromatin loci with IRF1 binding profiles for genes selected in Fig 3F. Called IRF1 peaks at the promoter are highlighted by red boxes.

To test genome‐wide binding of IRF1, we sequenced libraries derived from IRF1 ChIP in WT and IRF1 KO for ESCs and EpiLC in replicates (Dataset EV8). Lack of peak calling by MACS2 for IRF1 KO samples confirmed specificity of the used antibody (Fig EV4B). Peaks in ESCs (which have low IRF1 levels) still differed from KO conditions (Fig EV4B). Qualitatively, binding sites of IRF1 did not drastically change in differentiation (71 sites with FDR < 0.05). Quantitatively, IRF1 bound sites are weakly marked already in ESCs, and binding signal increases upon differentiation in EpiLCs (Fig 4A).

We compared IRF1 binding sites to those of OCT4 as a hallmark of pluripotency‐related sites (Buecker *et al*, 2014a; Data ref: Buecker *et al*, 2014b). 942/1770 of IRF1 sites overlapped with OCT4 found under either ESC, EpiLC or both conditions (Fig EV4C). The majority of the sites bound by both OCT4 and IRF1 are not changing their OCT4 binding in differentiation. More EpiLC‐specific OCT4 sites overlap with IRF1 sites (261) than ESC‐specific ones (19; Fig 4B). One example of such a EpiLC‐specific site is the *eOct6* enhancer used as reporter in CRISPR‐KO screen (Appendix Fig S2B).

*Irf1* KO EpiLCs express lower levels of *Oct6* than control, and *Irf1* scored as a screenhit in the set‐up which included Oct6 enhancer activity as a readout. *Irf1* KO EpiLCs are shifted along the *eOct6* enhancer controlled mCherry expression axis and showed lower fluorescent values in FACS analysis (Appendix Fig S2A, C, and E). This effect was already present in ESCs, here also, the basal e*Oct6* enhancer activity was reduced. GFP levels controlled by the e*Tbx3* enhancer were not affected (Appendix Fig S2A, C, and E).

We tested whether *eOct6* enhancer activity can be rescued in *Irf1* KO background by IRF1 expression. We expressed doxycycline‐inducible *Irf1* from cDNA in the dual reporter cell lines. Induction with doxycycline under ESCs conditions induced higher IRF1 expression than endogenous expression levels in EpiLCs (Appendix Fig S2D). The *eOct6* reporter was also more active in ESCs compared with WT and *Irf1* KO (Appendix Fig S2C). OCT6 protein levels were not increased in doxycycline‐treated ESCs, indicating that even high enhancer activity cannot drive gene expression in a non‐permissible chromatin environment (Appendix Fig S2D). Doxycycline treatment of reporter cells during differentiation also increased IRF1 levels and *eOct6* activity to higher levels compared with control differentiation. *eTbx3* always reported the differentiation status of the cells, independent of doxycycline treatment or *Irf1* background.

Doxycycline induced very strong expression of IRF1 and did not recapitulate normal expression levels. Therefore, we additionally used the *eOct6* enhancer to control IRF1 expression to enable differentiation‐induced IRF1 expression during the exit from naive pluripotency. In cells transfected with this construct, IRF1 was already increased in ESCs at the protein level (Appendix Fig S2F), most likely due to low enhancer activity in ESCs. This weak expression corresponded to an already increased *eOct6* enhancer controlled mCherry signal in ESCs (Appendix Fig S2E). In EpiLCs, IRF1 expression levels were fully restored or slightly increased in comparison with endogenous levels (Appendix Fig S2F). This correlated with increased *eOct6* enhancer activity driving mCherry. Throughout all rescue conditions, *eTbx3* controlled GFP levels were not influenced and changed according to differentiation status (Appendix Fig S2C and E).

We identified a canonical IRF1 binding motif (gAAAgtGAAA) in the *eOct6* enhancer itself. To test whether this motif is necessary for enhancer activity, we mutated 11 nucleotides within the IRF1 motif (from here referred to as *eOct6‐∆Irf1*). We generated cell lines in WT and *Irf1* KO background which contain *eOct6‐∆Irf1* controlling mCherry expression. In addition, we included the wild‐type *eOct6* enhancer controlling GFP expression as an internal control (Appendix Fig S2G). As expected, the unmodified *eOct6* enhancer was activated in WT cells and to a lesser extent than in *Irf1* KO cells. However, with the IRF1 binding motif mutated, the *eOct6* enhancer is activated independent of the presence or absence of IRF1, as *Irf1* KO shows similar activation levels compared with WT cells. The IRF1 binding motif mutated *eOct6‐∆Irf1* enhancer is still increasing activity in differentiation into EpiLCs, stressing that IRF1 is not the sole regulator of *eOct6* activity, but that a combination of factors is involved in the activation of the enhancer. In conclusion, IRF1 directly regulates *eOct6* enhancer activity dependent on the IRF1 binding motif, but IRF1 is not the sole regulator of this enhancer.

### IRF1 binding sites are involved in viral defense

As IRF1 binding showed little overlap with cell state‐specific OCT4 binding, we asked whether the IRF1 targets in EpiLCs are also activated in the innate immune response. Therefore, we compared ESC and EpiLC IRF1 binding sites to those in BMDMs, before and after IFNɣ stimulation (Data ref: Langlais *et al*, 2016b; Fig 4C). 64% of ESC/EpiLC IRF1 binding sites are also bound by IRF1 in BMDMs, either stimulated or unstimulated with IFNɣ. We compared genomic annotations between the sites shared between BMDMs and EpiLCs. Shared sites are located more often at promoter regions, while stem cell‐specific sites are located in intergenic regions, including enhancer sites (Fig EV4D).

GO term enrichment across all stem cell IRF1 binding sites confirmed integration into biological processes such as defense response to other organisms and innate immunity processes (Fig 4D).

Excitingly, in EpiLCs, IRF1 directly binds to promoters of genes that code for core components of the interferon response: For example, *Ddx58/Rig‐I* and its ligand‐specific sentinel *Ddx60* (Oshiumi *et al*, 2015) showed increased IRF1 binding at the promoter in differentiation (Fig 4E). Furthermore, both genes are upregulated during the transition from ESC to EpiLCs (Fig 3E), and overexpression of IRF1 in ESCS leads to induction of these genes (Fig 3F), establishing both genes as direct targets of IRF1 in the exit from naive pluripotency. DDX58/RIG‐I is known as a sensor of viral RNA, including for vesicular stomatitis virus (Kato *et al*, 2005; Yoneyama *et al*, 2005; Kell & Gale, 2015). Other examples include *Trim56* and *Rsad2/Viperin*, which are also bound and regulated by IRF1 and well‐established components of innate immune response (Fig 4E).

Taken together, IRF1 directly regulates a subset of ISGs. Many of these targets are conserved between early embryonic and somatic cell types.

### Intrinsic expression of IRF1 defends formative pluripotent cells against viral infections

**Figure 5:**
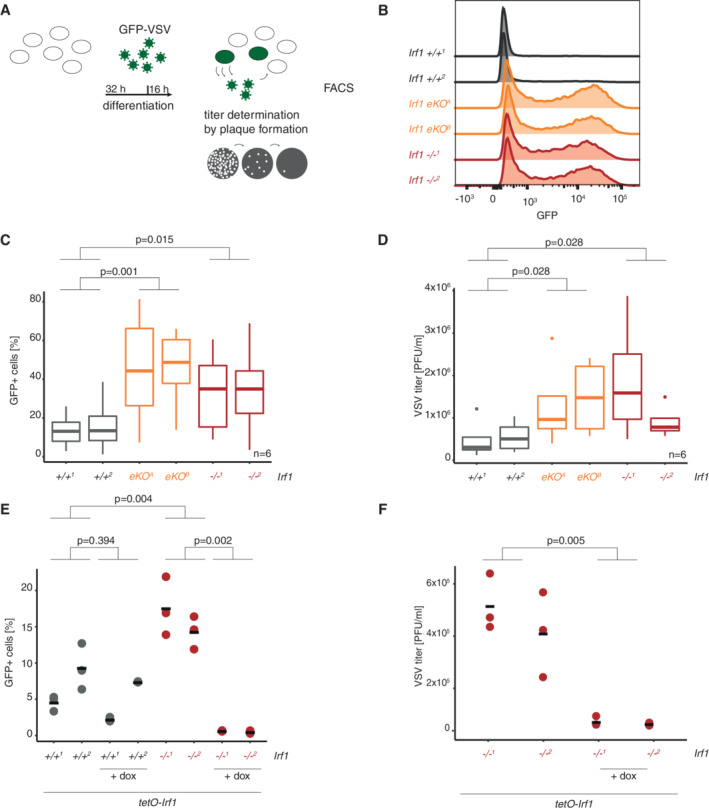
IRF1 protects EpiLCs from viral infection AExperimental strategy to analyze viral infection in ESC to EpiLC transition. Cells were differentiated for 32 h and then infected with GFP‐VSV. GFP^+^ cells were scored by FACS and titers were determined by plaque formation assays.BRepresentative FACS profiles of GFP‐VSV infected *Irf1* ^+/+^, *Irf1* enhancer KO and *Irf1* ^−/−^ cells. See Fig 5C for quantification.CQuantification of GFP^+^ cells after GFP‐VSV infection. *P*‐values were calculated per Wilcoxon test. *n* = 6 biological replicates. The central band shows the median, the box 25^th^ and 75^th^ percentiles, whiskers show 1.5*IGR (interquartile range).DViral titer as determined by plaque formation assays. *P*‐values were calculated per Wilcoxon test. *n* = 6 biological replicates. The central band shows the median, the box 25^th^ and 75^th^ percentiles, whiskers show 1.5*IGR (interquartile range).EQuantification of GFP^+^ cells after GFP‐VSV infection, IRF1 doxycycline‐inducible overexpression in *Irf1* ^+/+^ and *Irf1* ^−/−^ cells, without and with doxycycline induction. *P*‐values were calculated per Wilcoxon test. *n* = 3 biological replicates, black horizontal lines show the mean of the data.FViral titer as determined by plaque formation assays, IRF1 doxycycline‐inducible overexpression in *Irf1* ^−/−^ cells, without and with doxycycline induction. *P*‐values were calculated per Wilcoxon test. *n* = 3 biological replicates, black horizontal lines show the mean of the data.

**Figure EV5:**
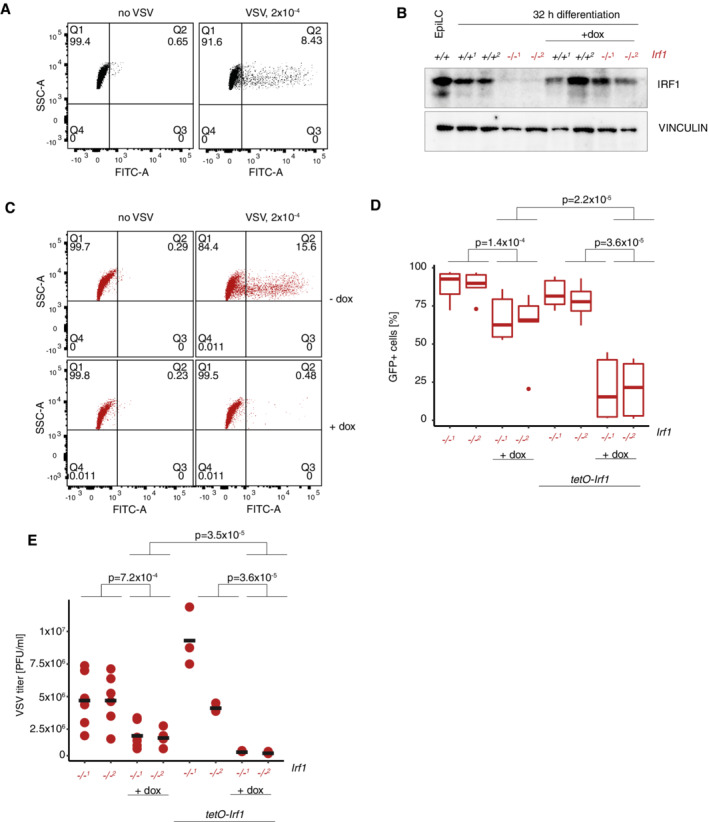
IRF1 in EpiLCs is required for protection against viral infection ARepresentative FACS profiles of cells infected with GFP‐VSV. Cells were scored according to FITC‐A signal.BWestern blot analysis of IRF1 rescue with doxycycline‐inducible *Irf1* construct. VINCULIN is used as loading control. All rescue samples were analyzed in two biological replicates.CRepresentative FACS profiles of IRF1 doxycycline‐inducible overexpression in cells infected with GFP‐VSV, without and with doxycycline treatment. Cells were scored according to FITC‐A signal.DQuantification of GFP^+^ cells after GFP‐VSV infection (higher virus concentration, see Methods), *Irf1* ^−/−^ and doxycycline‐inducible overexpression in *Irf1* ^−/−^ cells, without and with doxycycline induction. *P*‐values were calculated per Wilcoxon test. *n* = 6 biological replicates. The central band shows the median, the box 25^th^ and 75^th^ percentiles, whiskers show 1.5*IGR (interquartile range).EViral titer as determined by plaque formation assays, *Irf1* ^−/−^ and IRF1 doxycycline‐inducible overexpression in *Irf1* ^−/−^ cells, without and with doxycycline induction, as in panel (D). *P*‐values were calculated per Wilcoxon test. *n* = 3 biological replicates for rescue, *n* = 6 for non‐rescue conditions. Black horizontal lines show the mean of the data.

ISGs play a major role in defense against viral infection, and IRF1 is a direct regulator of a subset of ISGs. Therefore, we investigated whether IRF1‐mediated ISG expression in EpiLCs influences susceptibility to viral infection. We first differentiated WT and KO ESCs for 32 h to allow robust induction of IRF1 expression under WT conditions (Fig 5A). We then treated the differentiated cells for 16 h with vesicular stomatitis virus expressing GFP (VSV‐GFP). VSV has broad tissue tropism, suggesting that viral entry is dependent on ubiquitous mechanisms (Finkelshtein *et al*, 2013). GFP expression allows direct readout of infection rates by FACS analysis of GFP‐positive cells (Figs 5B, C and E, and EV5A, C and D). Cells were infected with a low multiple of infection (MOI) to establish a multicycle infection in *Irf1* WT cells. Strikingly, the percentage of infected cells significantly increased in *Irf1* KO (Fig 5B and C). Furthermore, deletion of the EpiLC‐specific enhancer controlling *Irf1* expression also increased viral infection compared with WT cells (Fig 5B and C).

Next, we quantified the viral replication ability by determining the viral titers in the supernatant of infected cells (Fig 5A). Full KO of *Irf*1, but also reduced levels of IRF1 by enhancer KO, increased viral titers (Fig 5D). We tested whether reexpression of IRF1 from a doxycycline‐inducible construct could reduce the infection levels (Fig EV5B and C). Indeed, IRF1 overexpression in differentiated *Irf1* KO significantly lowered infection rates (Fig 5E) and viral titers (Fig 5F). In addition, we challenged *Irf1* KO cells with and without IRF1 rescue with higher viral concentrations (Fig EV5D and E). Here, doxycycline treatment alone had an effect, but the reduction in the percentage of infected cells and viral titers was more pronounced in IRF1 overexpression conditions, arguing for a specific effect of exogenous IRF1 expression. The same effect has been described in somatic cells (Pine, 1992). We conclude that intrinsic upregulation of IRF1 is one of the defense mechanisms of EpiLCs against viral infections.

## Discussion

In this study, we provide evidence that the pluripotency network ensures constitutive expression of the transcription factor IRF1. This expression in turn is required for intrinsic expression of a subset of ISGs, and the expression of these ISGs defends formative cells against viral infection.

We performed a pooled CRISPR KO screen to identify factors whose deletion impairs the activation of the formative pluripotency gene regulatory network. For this, we used the expression dynamics of a dual reporter system to identify cells which are showing expected behavior—are unimpaired in differentiation—or those who are impaired in differentiation. In contrast to other CRISPR screens that for example depend on proliferation of cells over prolonged time frames, our screening set‐up is more sensitive to fluctuation due to noise. We counteracted these issues by focusing on a smaller library selected for nuclear factors, which could miss important factors such as metabolic regulators involved in differentiation (Moussaieff *et al*, 2015). Nevertheless, we identified several factors such as *Fgfr1* and *Zic3* which have already been studied in the context of the exit from naive pluripotency, stressing the validity of this approach (Molotkov *et al*, 2017; Yang *et al*, 2019).

We infected cells with a low MOI to ensure single knockout of one gene per cell. However, recent studies have shown the markable redundancy ensuring differentiation from naive to formative pluripotency: knockout of single factors delays, but not completely abolishes differentiation (Lackner *et al*, 2021). Differentiation is only abolished by simultaneous genetic deletion of three complementary drivers (Kalkan *et al*, 2019). Furthermore, the naive pluripotency network is supported by functionally overlapping factors of the same orphan nuclear receptor family (Festuccia *et al*, 2021). We therefore hypothesize that cooperativity and redundancy between two or even more factors are ensuring proper execution of this cell state transition, and that screening approaches should be adapted to this in the future.

We identified *Irf1* in our CRISPR KO screen as influencing the reporter activity of an enhancer region of *Oct6*, which we used as a proxy for the establishment of formative pluripotency. However, IRF1 deletion does not drastically change gene expression of other formative markers such as *Otx2*, *Fgf5*, and *Dnmt3a/b*, indicating that observed change in reporter activity is not strictly a readout of cell state. IRF1 is directly interacting with the chosen enhancer region through a canonical IRF1 chromatin binding motif. *Oct6* gene expression is reduced in the absence of IRF1. *Oct6* belongs to the family of POU TFs that share a highly similar DNA recognition motif. In fact, it was shown that OCT6 can replace OCT4 during reprogramming into human iPS cells due to the similarity in DNA motif binding (Kim *et al*, 2020). OCT6 and OCT4 occupy many of the same sites in epiblast stem cells (EpiSCs), and it is therefore not surprising that lower levels of OCT6 have only minor effects in the exit from naive pluripotency (Matsuda *et al*, 2017). Furthermore, deletion of *Oct6* in the mouse does not severely impact embryogenesis, again probably due to functional redundancy among different members of the POU transcription factor family (Bermingham *et al*, 1996; Kim *et al*, 2021).

Redundancy seems to be present not just in naive pluripotency, but also among many formative pluripotency regulators: The transcription factor OTX2 is sufficient to drive gene changes associated with formative pluripotency, but its deletion only has limited effect on the expression of formative genes (Yang *et al*, 2014; Buecker *et al*, 2014a). Misregulation of OCT6 could therefore also be compensated by other members of the formative gene regulatory network. While OCT6 has been studied mostly in the context of development and neurogenesis, it might have additional roles upon interferon stimulation. OCT6 itself is upregulated by interferons in macrophages and other cell types; however, its role in interferon signaling is unclear (Hofmann *et al*, 2010).

Importantly, *Irf1* itself is directly regulated by the formative pluripotency network through an enhancer, which is marked by activating chromatin marks as well as OCT4 and OTX2 in formative EpiLCs. This enhancer is stem cell specific as it does not get activated in stimulated BMDMs (Lara‐Astiaso *et al*, 2014). A subset of ISGs is differentially expressed in the exit from naïve pluripotency. IRF1 activates EpiLC‐specific ISGs, but so far, it is unclear what drives ESC‐specific ISG expression. ISG expression in early embryonic tissues, iPS or also somatic stem cells is conserved in mouse, pig, chimpanzee, and human (Wu *et al*, 2018; Shi *et al*, 2020). This expression is independent from viral stimulation and was previously only described as intrinsic (Wu *et al*, 2018) or as a consequence of endogenous retroviral expression observed in hypomethylated stem cells (Grow *et al*, 2015).

Wu *et al* (2018) showed that ISG expression in human stem cells can protect these cells from viral infection. Here, we show that similar mechanisms are at play in mouse stem cells, as IRF1 is a key factor required for the activation of several ISGs. Of note, the expression of ISGs is a physiological and constitutive property of differentiating cells, as we observed these relationships in the absence of viral infection. It has also been shown in hepatocytes that constitutive IRF1 expression maintains ISG expression and confers immediate defense against virus (Yamane *et al*, 2019). The strong protection against viral infection suggests that combined action of the ISGs regulated by IRF1 is required for viral defense. The naive/formative culture system recapitulates changes occuring at the time of implantation of the embryo into the uterus. We therefore speculate that prophylactic priming of the defense system could be crucial at a vulnerable moment in development, as implantation could be a risk for viral exposure. Interestingly, the upregulation of IRF1 in the embryo is transient and *Irf1* expression is already reduced right after implantation. We therefore speculate that *Irf1* expression might only be needed at this very short time period when the epiblast first encounters the mother's uterus.

**Figure 6:**
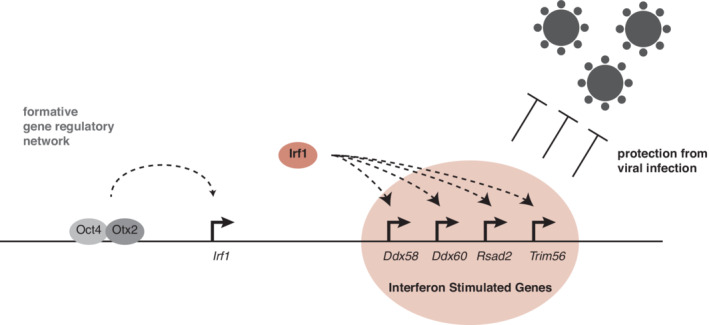
The formative pluripotency network regulates IRF1, which is required for defense against viral infections The formative pluripotency network directly activates Irf1 expression during the exit from naïve pluripotency through an EpiLC‐specific enhancers. IRF1 in turn activates a subset of ISGs. This proactively sets the machinery for defense against viral infections in place. In the presence of IRF1, cells therefore are stronger protected against viral infections.

We show here that the formative pluripotency network itself controls the expression of a subset of ISGs through upregulation of IRF1, and thereby protects this stage from viral infection (Fig 6). Robustness against viral infection appears to be an important property of proliferating stem cells and is controlled by the specific gene regulatory network that safeguards the stem cell state itself.

## Materials and Methods

### Reagents and Tools table

**Table:** 

Reagent/Resource	Reference or Source	Identifier or Catalog Number
**Experimental Models**		
mESC R1	Buecker *et al* (2014a)	N/A
mESC E14Tg2a	Acampora *et al* (2013	N/A
**Recombinant DNA**		
pB‐eOct6‐mCherry‐Puro	This study	N/A
pB‐e/Oct6‐‐GFP‐‐Neo	Thomas *et al* (2021)	N/A
pB‐eOct6ΔIrf1motif‐mCherry‐Puro	This study	N/A
pB‐eOct6ΔIrf1motif_extended‐mCherry‐Puro	This study	N/A
pB‐eTbx3‐dGDP‐Hygro	This study	N/A
pX330‐U6‐Chimeric_BB_CBh_hSpCas9	Addgene Plasmid	#42230
pX330‐U6‐Chimeric_noCas9	This study	N/A
pB‐transposase	System Biosciences Cat	#PB210PA‐1
PB‐gRNA‐Puro	Addgene	#121121
PB‐TetON‐dual‐SunTag‐Hygro	Addgene	#121119
PB‐CA‐rtTA Adv	Addgene	#20910
pB‐Oct6‐Irf1‐Neo	This study	N/A
PB‐tetO‐Irf1‐Neo	This study	N/A
PB‐tetO‐Irf1‐Blasti	This study	N/A
**Antibodies**		
GAPDH (WB 1:10,000)	Santa Cruz	sc‐365062
IRF1 (IF 1:200, WB 1:1,000)	CST	#8478
OTX2 (WB 1:1,000)	R&D Systems	AF1979
VINCULIN (WB 1:5,000)	Santa Cruz	sc‐73614
OCT6/Pou3F1 (WB 1:1,000)	Sigma/Aldrich	MABN738
Phospho‐STAT3 (Tyr705) (3E2) (WB 1:1,000)	CST	#9138
**Oligonucleotides and sequence‐based reagents**		
CRISPR KO library	This study	Dataset EV1
PCR primers	This study	Dataset EV2
pPCR primers	This study	Dataset EV2
CRISPR guides (validations)	This study	Dataset EV2
**Chemicals, enzymes and other reagents**		
poly‐L‐ornithine hydrobromide	Sigma‐Aldrich	P4638
laminin	Sigma‐Aldrich	L2020
human Plasma Fibronectin Purified Protein	Sigma‐Aldrich	FC010‐10MG
AlbuMAX^TM^ II Lipid‐Rich Bovine Serum Albumin	Fisher Scientific	11021‐029
MACS NeuroBrew‐21 with Vitamin A (Miltenyi Biotec)	Miltenyi Biotec	130‐093‐566
MEM NEAA	Thermo Fisher Scientific	12084947
Penicillin‐Streptomycin	Thermo Fisher Scientific	15070063
Sodium Pyruvate	Thermo Fisher Scientific	15070063
2‐Mercaptoethanol	Fisher Scientific	11508916
CHIR‐99021	Selleck Chemicals	S1036
PD0325901	Selleck Chemicals	S1263
hLIF	Provided by the VBCF Protein Technologies Facility	https://www.viennabiocenter.org/facilities/
Recombinant Human FGF‐basic	peprotech‐eubio	100‐18B‐100μG
KnockOutTM Serum Replacement	Fisher Scientific	Gibco™ 10828028
Trypsin‐EDTA solution	Sigma‐Aldrich	T3924‐100ML
FSC	Sigma‐Aldrich	F7524‐500ML
ruxolitinib	Invivogen	INCB018424
Lipofectamine® 2000	Invitrogen	10696153
G415 Disulfate solution	Sigma‐Aldrich	G8168‐10ML
doxycyclin	Sigma‐Aldrich	D9891‐1G
hygromycin B	Sigma‐Aldrich	10843555001
puromycin	Invivogen	ant‐pr‐1
HEPES‐buffered saline, pH 7.0 (2× for transfection)	VWR	J62623.AK
SpeedBeads™	Sigma‐Aldrich	GE45152105050250
pepGOLD TriFastTM reagent	VWR	130‐2010
cOmpleteTM Protease Inhibitor Cocktail	Sigma‐Aldrich	11836145001
DAPI	Sigma‐Aldrich	D9542
RIPA lysis buffer	Merck	20‐188
DMEM high glucose	Sigma‐Aldrich	D6429
Avicel‐RC591	FMC BioPolymer	N/A
**Software**		
bbmap	https://jgi.doe.gov/data‐and‐tools/software‐tools/bbtools/	N/A
ChIPpeakAnno (3.24.2)	http://www.bioconductor.org/packages/release/bioc/html/ChIPpeakAnno.html	N/A
deeptools 3.5.1	https://deeptools.readthedocs.io/en/develop/	N/A
htseq/0.11.2‐foss‐2018b‐python‐3.6.6	https://htseq.readthedocs.io/en/master/	N/A
MAGeCK 0.5.9.	https://sourceforge.net/p/mageck/wiki/Home/	N/A
Nextflow/21.04.1 nf‐core	https://nf‐co.re	N/A
nf‐core/chipseq v1.2.2	https://nf‐co.re/chipseq/1.2.2/usage	N/A
star/2.7.1a‐foss‐2018b	https://github.com/alexdobin/STAR	N/A
tophat/2.1.2‐foss‐2018b	http://ccb.jhu.edu/software/tophat/index.shtml	N/A
R4.0.4	https://cran.r‐project.org	N/A
ggplot2 (3.3.5)	https://cran.r‐project.org/web/packages/ggplot2/index.html	N/A
DESeq2 (1.30.1)	https://bioconductor.org/packages/release/bioc/html/DESeq2.html	N/A
pheatmap (1.0.12)	https://cran.r‐project.org/web/packages/pheatmap/index.html	N/A
GREAT (4.0.4)	http://great.stanford.edu/public/html/	N/A
ImageJ	https://imagej.net	N/A
**Other**		
BD FACSMelody Cell Sorter	BD Biosciences	N/A
BD FACSAria	BD Biosciences	N/A
BD FACSFortessa	BD Biosciences	N/A
Zeiss Axio Oberserver Z1	Zeiss	N/A
llumina HiSeqV4 SR50	Illumina	N/A
Illumina NextSeq550 PE75	Illumina	N/A
Chemidoc Touch	Bio‐Rad	N/A
Bioanalyser	Agilent	N/A
μ‐Slide 8 Well Chambered Coverslip	ibidi	#80827
AMPure XP beads	Beckman Coulter	A63880
PVDF Transfer Membranes	Thermo Scientific	#88518
NucleoSpin PCR and Gel Purification Kit	Macherey‐Nagel	#15559212
NEBNext Library Quant Kit for Illumina	NEB	E7630S
SensiFast^TM^ cDNA Synthesis kit	Bioline	67.BIO‐65054
SensiFASTTM SYBR® No‐ROX kit	Bioline	67.BIO‐98020
3’ mRNA Seq Library Prep Kit	Lexogen	No. 081.96
sparQ DNA Library Prep Kit	Quanta Bio	#95191‐096
Alkaline Phosphatase Detection Kit	Sigma‐Aldrich	SCR004
Bio‐Rad Protein Assay	BioRad	#5000006

### Methods and Protocols

#### ESC maintenance and differentiation conditions

Murine embryonic stem cells (E14Tg2a for screening, R1 for all other cell lines) were cultured and differentiated as described in Thomas *et al* (2021). For maintenance, cells were grown on CELLSTAR^®^ 6/12 wells coated with first poly‐L‐ornithine hydrobromide (6 μg/ml in PBS, 1 h at 37°C, sigma P4638) and then laminin (1.2 μg/ml in PBS, 1 h at 37°C, Sigma L2020). Cultures were tested regularly for mycoplasma contamination. For differentiation and viral infection, plates were coated with fibronectin (Human Plasma Fibronectin Purified Protein, Sima‐Aldrich, 5 μg/ml in PBS, 1 h at RT).

Cells were cultured in base medium HyClone DMEM/F12 without Hepes (Cytiva) with 4 mg/ml AlbuMAX™ II Lipid‐Rich Bovine Serum Albumin (GIBCO™), 1× MACS NeuroBrew‐21 with Vitamin A (Miltenyi Biotec), 1× MEM NEAA (GIBCO™), 50 U/ml Penicillin–Streptomycin (GIBCO™), 1 mM Sodium Pyruvate (GIBCO™), and 1× 2‐Mercaptoethanol (GIBCO™). For 2i + LIF culturing conditions, base medium was supplemented with with 3.3 mM CHIR‐99021 (Selleckchem), 0.8 mM PD0325901 (Selleckchem), and 10 ng/ml hLIF (provided by the VBCF Protein Technologies Facility, https://www.viennabiocenter.org/facilities/). For differentiation, base medium was supplemented with 12 mg/ml Recombinant Human FGF‐basic (PEPROTECH) and KnockOutTM Serum Replacement (1:100, GIBCO™).

For splitting, cells were treated with 1× Trypsin–EDTA solution (sigma T3924) at 37°C until cells detached. Trypsinization was stopped with 2i + LIF medium with 10% FSC (Sigma F7524), cells were harvested by centrifugation at 300 *g* for 3 min, resuspended in 2i + LIF and seeded in appropriate ratios.

For differentiation, cells were seeded a day prior on fibronectin‐coated plates in 2i + LIF medium (100 k per 12 well, 200 k per 6 well). The next day, differentiation was started by removing the medium, two washes of the attached cells with PBS, and the addition of differentiation medium. Cells were collected as EpiLCs after 48 h.

For JAK inhibition experiments, cells were treated as indicated on the onset of differentiation with 1.5 μM ruxolitinib (Invivogen).

#### Generation of reporter and screening cell lines

Cells used for the dual fluorescent reporter system and CRISPR screening were based on an Otx2^flox/−^; R26^CreER/+^ ESC cell line (Acampora *et al*, 2013). Otx2 heterozygous expression does not impair ESC to EpiLC differentiation (Acampora *et al*, 2013). pB‐transposase, pB‐eOct6‐mCherry‐Puro and pB‐eTbx3‐dGFP‐Hygro were lipofectamine transfected. After selection, two clonal cell lines with clear distinction of ESC/EpiLC states in FACS analysis were selected. Lentivirus containing Cas9‐Blasticidin was used for constitutive CAS9 expression. Neomycin resistance derived from the original present LacZ allele was removed by CRISPR KO.

For pB‐eOct6ΔIrf1‐mCherry‐Puro, the mutated Irf1 binding sites were placed on overlapping primers, and the Oct6 enhancer was PCR amplified and the plasmid constructed with three fragment Gibson Assembly. pB‐transposase, pB‐eOct6ΔIrf1‐mCherry‐Puro and pB‐eOct6‐mCherry‐Neo were co‐transfected into ESCs with lipofectamine.

#### Generation of KO and rescue cell lines

Knockouts of coding sequences or enhancer regions were performed with dual CRISPR sgRNA KO. For this, CRISPR sgRNAs were inserted into pX330‐U6‐Chimeric_noCas9 (for cell lines already expressing Cas9) or pX330‐U6‐Chimeric_BB_CBh_hSpCas9 using BbsI (NEB) directed cloning. sgRNA plasmid combinations and substoichiometric amounts of dsRed plasmid were cotransfected by Lipofectamine® 2000 (Invitrogen) transfection (375 ng each sgRNA plasmid, 50 ng dsRed, 5 μl lipofectamine in total 100 μl DMEM/F12) into cells seeded on 12‐well plates 1 day prior. Two to three days after transfection, dsRed^+^ cells as proxy for sgRNA transfection were FACS‐sorted (BD FACSMelody Cell Sorter) as single cells on 96‐well plates coated with fibronectin. Successful genome editing was confirmed by genotyping PCRs and Western blotting where applicable.

For doxycycline‐inducible reexpression of Irf1 in Irf1 KO cell lines, we cloned Irf1 cDNA under tetO control with either Neomycin or Blasticidin selection cassettes. Transfected and selected cells were analyzed as pools without clonal selection. Dox treatment (1 μg/ml) was performed for 48 h, in ESC or EpiLC medium as indicated.

For differentiation‐induced rescue of Irf1 expression, we cloned pB expression constructs containing Irf1 cDNA under control of the Oct6 enhancer. This resulted in low Irf1 expression levels in ESCs and upregulation of Irf1 upon differentiation. Transfection of pB‐Oct6enh‐Irf1‐Neo was performed with lipofectamine transfection as described for reporter constructs, albeit with lower DNA amounts (250 ng pB plasmid). After neomycin selection (400 μg/ml, G415 Disulfate solution, sigma G8168‐10ML), pools of transfected cells were differentiated and analyzed by FACS and Western blotting.

#### IRF1 overexpression with SunTag system

Overexpression of Irf1 from the endogenous locus was based on a SunTag overexpression system published in Heurtier *et al*, 2019. Following plasmids were ordered from Addgene: 121121 PB‐gRNA‐Puro, 121119 PB‐TetON‐dual‐SunTag‐Hygro, 20910 PB‐CA‐rtTA Adv. Ten microgram PB Transposase, 500 ng PB‐TetON‐dual‐SunTag‐Hygro and 500 ng PB‐CA‐rtTA were transfected into R1 ESCs by electroporation (500 μF, 240 V, 4 mm). Cells were treated with doxycycline (1 μg/ml) and hygromycin (400 μg/ml). After 5 days, BFP^+^/GFP^+^ double positive cells were selected by FACS. Cells were grown in the absence of doxycycline and hygromycin and BFP^−^/GFP^−^ double negative cells were selected by FACS. Single clones were selected and tested by FACS and Western blotting against Cas9 to select for response to doxycycline treatment.

sgRNAs targeting the Irf1 promoter were designed in benchling, which is based on (Hsu *et al*, 2013) and cloned into PB‐gRNA‐Puro by BbsI directed cloning. The sgRNA plasmid and PB transposase were lipofectamin transfected into parental SunTag cell lines and selected by puromycin (2 μg/ml, Invivogen) treatment. Irf1 upregulation after dox treatment was confirmed by qPCR and Western blotting.

#### CRISPR screening

CRISPR screening was based on (Michlits *et al*, 2017), but without using clonal dilution steps. DNA pools of indicated subpools were combined according to sgRNA number and CaCl Hepes transfected into PlatE cells, including helper plasmid. Twenty‐four hours after transfection, medium was exchanged to 2i + LIF. Virus containing 2i + LIF was harvested in two batches over the next 36 h. Both batches were pooled and frozen. MOI was determined by determining infection efficiency without selection.

#### Execution of CRISPR screen

The screen was performed with two independent replicates with different screening cell lines. To reach sufficient coverage of the library, we infected 100 million cells at a MOI of 0.3 by seeding 7.5 million cells per 15 cm dish coated with fibronectin (Dataset EV1). Starting library representation was accessed by infecting cells without Cas9 (R1) in parallel. In addition, no selection, kill and no sgRNA library control plates were prepared. Usage of fibronectin coated plates allows to omit polybrene addition during infection. As soon as cells were attached (2 h), virus was added to the cells without additives or medium exchanges (day1). The next day, selection with neomycin was started and continued for 2 days. This short but stringent selection (400 μg/ml, G415 Disulfate solution, sigma G8168‐10ML) resulted in complete elimination of nonresistant cells. From now on, each day cells were either splitted or medium was exchanged. On day 4, the kill control was dead and 30 mio library infected cells were seeded on 15 cm PLOL plates with 7.5 mio per plate without selection. Empty library controls were carried throughout the screen. On day 6 and 8, again 30 mio cells were seeded without selection, allowing time for depletion of essential genes. On day 10, 60 mio cells were harvested and analyzed later as samples representing genes essential for ESC maintenance. For differentiation, 30 mio cells were seeded on fibronectin‐coated dishes (7.5 mio per 15 cm dish) In addition, extra dishes as 2i + LIF control and no sgRNA library controls were seeded. On day 11, differentiation was induced by 2 + iLIF medium removal, 2 washed in PBS and addition of differentiation medium. Starting time points for different plates were staggered to allow FACS analysis after 48 of differentiation for each plate.

On day 13, cells were harvested and sorted by BD FACSAria (BD Biosciences). For this, gates were set up using no sgRNA library control cells, which were processed in parallel. At least 60 mio cells were sorted in the unimpaired gate and all possible cells in the impaired gate (N1 = 64.7–10.3 mio, N2 = 71.5–5.6 mio cells). In addition, 60 mio cells for essential ESC and 60 mio for unsorted EpiLCs were harvested for each condition. The screen was performed as replicate using two independently generated cell lines.

#### gDNA isolation, PCR amplification, and NGS

CRISPR KO libraries were generated from collected cells as described by (Michlits *et al*, 2017), scaled according to the cell numbers used.

In brief, cell pellets were resuspended in SDS‐lysis buffer and incubated at 55°C overnight to lyse cells, followed by RNaseA treatment. Protein was precipitated by NaCl precipitation, the supernatant was then purified with phenol/chloroform. DNA was precipitated with isopropanol and dissolved in TE buffer. DNA was PacI digested for a total of 48 h and size selected with SpeedBeads™ (Millipore Sigma) in two rounds to enrich fragments < 2 kb.

CRISPR‐UMI cassettes were PCR amplified and multiplexed with 25 cycles of 1:1 KlenTag/Phusion PCR (Jena Bioscience and in house generated) using primers specified in Michlits *et al* (2017). Reactions were purified using the NucleoSpin PCR and Gel Purification Kit (Macherey‐Nagel). Samples were pooled after qPCR quantification, purified from 1.5% agarose, and quantified with NEBNext Library Quant Kit for Illumina (NEB). Sequencing was performed on Illumina HiSeqV4 SR50 (Dataset EV3, Dataset EV4).

#### RT‐qPCR analysis

ESC and EpiLC were directly lysed on the plate using pepGOLD TriFastTM reagent (Peqlab) according to the manufacturer's instructions. Five hundred nanogram of RNA were reversed transcribed to cDNA with the SensiFast™ cDNA Synthesis kit (Bioline) according to the manufacturer's instructions.

For qPCR, cDNA was 1:5 diluted and 0.5 μl were used per 10 μl qPCR reaction with SensiFASTTM SYBR® No‐ROX kit (Bioline) and 125 nM forward and reverse primer. Irf1‐specific primers were based on (Platanitis *et al*, 2019).

qPCRs were analyzed by ΔΔ*C*_T_ and normalized to ESCs samples of the corresponding cell lines. Statistical tests were performed as homoscedastic one‐sided *t*‐tests.

#### FACS analysis

ESC and EpiLC were harvested by trypsin treatment, which was stopped by 1:1 addition of 10% FSC. Samples were strained through 5‐ml polystyrene round‐bottom tubes with cell‐strainer caps (Falcon). For analytical purposes, the BD Fortessa was used, for cell sorting in high‐throughput BD FACSAria or in low‐throughput BD FACSMelody. Single cells were gated according to FFW and SSW scattering.

#### Quantseq

RNA QuantSeq was prepared according to the manufacturer's instructions (Lexogen 3' mRNA Seq Library Prep Kit). Five hundred nanogram RNA was used as starting material. qPCRs were used to multiplex samples and final libraries were quantified by NEBNext Library Quant Kit for Ilumina (#E7630S).

#### Chromatin immunoprecipitation

ChIP with IRF1 was performed as described in Thomas *et al* (2021). In brief, 3 mio cells were seeded on 15 cm fibronectin‐coated dishes, medium exchanged to 2i + LIF or differentiation the next day and cells harvested after 48 h.

For harvesting, cells were cross‐linked in 1% formaldehyde in PBS for 10 min. Cross‐linking was quenched in 0.125 M glycine. From now on, samples were kept at 4°C. Fixed cells were washed on the plate twice with PBS and scraped off in 0.01% Triton in PBS. Cells were harvested by 500 *g* 5 min centrifugation and flash‐frozen in liquid nitrogen. Cell pellets were resuspended in 5 ml LB1 (50 mM Hepes pH 7.5, 140 mM NaCl, 1 mM EDTA, 10% glycerol, 0.5% NP‐40, 0.25% TX‐100, 1 mM PMSF, 1× cOmpleteTM Protease Inhibitor Cocktail (Roche) by rotating vertically for 10 min at 4°C and harvested (5 min, 1,350 *g*, 4°C)). The pellet was resuspended in 5 ml LB2 (10 mM Tris pH 8. 200 mM NaCl, 1 mM EDTA, 0.5 mM EGTA1, mM PMSF, 1× cOmpleteTM Protease Inhibitor Cocktail (Roche)), 10 min vertical rotation, room temperature. Centrifugation was repeated and pellets were suspended in 1.5 ml LB3 (10 mM Tris–HCl pH 8, 100 mM NaCl, 1 mM EDTA 0.5 mM EGTA, 0.1% Na‐deoxycholate, 0.5% N‐lauroylsarcosine, 1 mM PMSF, 1× cOmpleteTM Protease Inhibitor Cocktail (Roche)) and 200 μl sonification beads (diagenode) in Bioruptor® Pico Tubes (diagenode). Sonification was performed for 13 cycles 30 s on/45 s off. Supernatant, but not the beads, were transferred to fresh tubes and cellular debris was pelleted at 16,000 *g* at 4°C. 1.1 ml were transferred to fresh tubes and 110 μl 10% triton to final 1% were added. Fifty microliter was saved as input and 1 ml was used for ChIP.

Antibody precipitation was performed with 5 μl IRF1 antibody overnight at 4°C with vertical rotation. Next day, Dynabeads protein G for Immunoprecipitation (Thermo Fisher Scientific) were used. One hundred microliter beads were washed in block solution (0.5% BSA in PBS) and incubated with chromatin/antibody solutions for at least 2 h. Bound beads were washed five times in cold RIPA wash buffer (50 mM Hepes pH 7.5, 500 mM LiCl, 1 mM EDTA, 1% NP‐40, 0.7% Na‐Deoxycholate), followed by three washes in TE + 50 mM NaCl. Bound fractions were eluted in 210 μl elution buffer (50 mM Tris pH 8.0, 10 mM EDTA, 1% SDS) for 15 min at 65°C. Supernatant was removed from the beads. Input samples were diluted with three volumes of elution buffer. Input and ChIP samples were decrosslinked at 65°C overnight.

One volume of TE was added to all samples as well as RNase A (0.2 mg/ml final) and incubated at 37°C for 2 h. Salt concentration was adjusted to final 5.25 CaCl_2_ with 300 mM CaCl_2_ in 10 mM Tris pH 8.0 and Proteinase K added to final 0.2 mg/ml. Digestion was performed at 55°C for 30 min. DNA was phenol‐chloroform extracted in Phase Lock GelTM tubes (Quantabio), ethanol precipitated and dissolved in H20.

ChIP was checked by performing qPCRs and calculating recovery of input (%).

Sequencing libraries were generated with sparQ DNA Library Prep Kit (Quanta Bio #95191–096) according to the manufacturer's instruction and using AMPure XP beads. Adapter contamination was cleaned up with an additional round of AMPure XP bead purification. Quality of the libraries were checked by Bioanalyser (Agilent) and final concentrations were determined with the NEB library Quant Kit.

#### Alkaline phosphatase staining

For AP staining, cells were seeded in different densities on gelatine‐coated plates. The next day, differentiation was performed for 72 h, controls were kept in 2i + LIF medium. All cells were placed back in 2i + LIF medium. After 2–4 day, colonies were stained with the Alkaline Phosphatase Detection Kit (Sigma‐Aldrich) according to the manufacturer's instructions.

#### Immunofluorescence

Ten k cells were seeded per chamber of a μ‐Slide 8 Well Chambered Coverslip (ibidi). Cells were differentiated for 48 h, washed in PBS and fixed with 4% PFA for 15 min at RT. Cells were washed 3× in PBST (0.1% Tween in PBS). Permeabilization was performed in 0.1% Triton‐X in PBS for 10 min at RT. Cells were washed 3× in PBST and blocked in 5% BSA in PBST for 30 min at 4°C. Primary antibody was diluted in blocking buffer and incubated overnight at 4°C. Cells were washed 5× in PBST and incubated with secondary antibody in blocking buffer for 1 h at RT. Cells were washed 3× in PBST, followed by 2× PBS washes. Nuclei were stained with 20 ng/ml DAPI (Sigma, D9542) for 10 min. Cells were washed 3× in PBS and stored in PBS at 4°C until imaging. Imaging was performed on a Zeiss Axio Oberserver Z1 microscope with 63× oil objective. Images were processed using ImageJ.

#### Western blot analysis

For Western blot analysis, cells were harvested by trypsin treatment. The cell pellet was washed once in PBS, after supernatant removal dry pellets were frozen. Cells were lysed in 1× RIPA lysis buffer (Merck, 20‐188), including 1× cOmpleteTM Protease Inhibitor Cocktail (Roche) and phosphatase inhibitors (1 mM NaF, 20 mM β‐glycerophosphate, 1 mM Na_3_VO_4_) for 1 h on ice. Cellular debris was removed by centrifugation (16,000 *g*, 10 min, 4°C). Protein was quantified using Bio‐Rad Protein Assay (5000006). Twenty‐five microgram protein per sample were separated on 10% Tris‐gylcine SDS polyacrylamid electrophoresis. Wet‐blotting was performed onto PVDF Transfer Membranes (Thermo Scientific #88518). Membranes were blocked in 5% milk in PBST. Primary antibodies were incubated overnight at 4°C, secondary for 2 h at RT. HRP coupled secondary antibodies were used for detecting with GE Healthcare LS ECL Select WB detection reagent. Images were acquired on a Chemidoc Touch (Bio‐Rad).

Note that ɑIRF1 primary detected two bands, both which were specific to IRF1. The ratio of these bands is dependent on the presence or absence of phosphatase inhibitor (Roche PhosSTOP) in lysis conditions.

#### Viral infection

2.5 × 10^4^ cells were seeded a day before the start of differentiation on 24‐well plates in 2i + LIF medium. Differentiation was induced as described before, as indicated cells were doxycycline treated at the same time. After 32 h, virus was added to the cells. For this, a VSV‐GFP stock with a titer of 1 × 10^8^ PFU/ml (determined on A549 cells) was diluted 1:20,000 or 1:5,000 in differentiation medium. Two hundred microliter of virus dilutions were added to the existing 500 μl of medium on the cells, resulting in estimated MOIs of 0.02 and 0.08 PFU/ml, respectively. Cells were incubated for an additional 16 h.Subsequently, supernatants were collected, cleaned from any cells by centrifugation and stored at −80°C until titration by plaque assays. EpiLCs were collected by trypsinization and the cell pellets were fixed in 4% PFA (Electron Microscope Sciences #1570) in PBS for 15 min. One milliliter of PBS was added to dilute the PFA, cells were collected by centrifugation and resuspended in 10% FCS in DMEM/F12 for FACS analysis.

#### Plaque assay

Vero cells were cultured in DMEM high glucose (Sigma‐Aldrich D6429) with Penicillin–Streptomycin (Thermo‐Fischer 15070063) and 10% FCS (Sigma‐Aldrich, F7524). They were seeded at 50% confluency on day 1 in 6‐well plates, so that they were > 90% confluent on day 2. On day 2, sequential dilutions of viral supernatants were made in DMEM without additives, ranging from 10^−3^ to 10^−6^. Vero cells were incubated with 400 μl of viral dilutions for 1 h at room temperature in technical replicates. Afterward, viral dilutions were removed and cells were overlaid with 2 ml of 0.4%, in DMEM high glucose with 4.2% FCS to stop cell growth (Matrosovich *et al*, 2006). Cells were incubated at 37°C, 5% CO_2_ for 24–30 h. Then, cells were fixed in 2% formaldehyde (Sigma‐Aldrich F8775) in PBS for > 30 min. Plates were washed in tap water and stained with Crystal Violet Staining Solution (0.005% Crystal Violet, 1% Formaldehyde, 1X PBS, 1% methanol). Plaques in suitable dilutions were manually counted, blinded toward sample identity. Titers were averaged from the technical replicates.

#### Data analysis

For CRISPR KO screening, reads were aligned to the library using custom scripts as published in Michlits *et al* (2017). Starting from count tables, analysis was performed with MAGeCK 0.5.9.2 and R 4.0.4. Plots were generated with ggplot2 (3.3.5).

QuantSeq RNA‐seq data was processed following Lexogen's standard pipeline on bluebee. Adapter contamination was removed with bbmap, mapped using STAR and counted with HTScount. Downstream analysis was performed in R using DESeq2 (1.30.1) and pheatmap (1.0.12).

QuantSeq RNA data were used to also analyze repeat elements as in Percharde *et al* (2017). For this, Tophat2 with the setting g‐1 was used to retain multimappers mapped to one random location, repeat elements were counted using a custom gtf file. Downstream analysis was performed in R using DESeq2 (1.30.1) and pheatmap (1.0.12).

For ChIP‐Seq analysis, the Nextflow/21.04.1 nf‐core/chipseq v1.2.2 pipeline was used with mm10 as reference genome. Profiles and heatmaps were generated with deeptools 3.5.1. ChIP data were analyzed with ChIPpeakAnno (3.24.2) and GO term were determined with GREAT (4.0.4) analysis (McLean *et al*, 2010).

ISGs were defined based on (Wu *et al*, 2018).

## Author contributions

**Merrit Romeike:** Conceptualization; formal analysis; investigation; writing—original draft; writing—review and editing. **Stephanie Spach:** Data curation; investigation. **Marie Huber:** Data curation; investigation. **Songjie Feng:** Data curation; investigation. **Gintautas Vainorius:** Resources; methodology. **Ulrich Elling:** Resources; methodology. **Gjis A Versteeg:** Investigation; methodology. **Christa Buecker:** Conceptualization; supervision; funding acquisition; writing—original draft; project administration; writing—review and editing.

In addition to the CRediT author contributions listed above, the contributions in detail are:

CB and MR conceived and designed the study. MR, SS, and MH generated and validated cell lines and carried out experiments. SF generated and validated enhancer KO cell lines. MR carried out the screen. MR and GV analyzed screening data, with input from UE. GAV provided VSV‐GFP and expertise in viral infections and titer determination. MR analyzed all sequencing data. MR and CB wrote the manuscript, with input from all co‐authors. CB supervised the project.

## Disclosure and competing interests statement

The authors declare that they have no conflict of interest.

## Supporting information




Appendix

Expanded View Figures PDF

Dataset EV1

Dataset EV2

Dataset EV3

Dataset EV4

Dataset EV5

Dataset EV6

Dataset EV7

Dataset EV8

## Data Availability

The NGS datasets produced in this study are available in the European Nucleotide Archive, https://www.ebi.ac.uk/ena/browser/home and assigned the identifier PRJEB53216 (https://www.ebi.ac.uk/ena/browser/view/PRJEB53216?show=reads). Processed data have been included as extended view Datasets  in this manuscript.
