# Supplementary material for: Transient upregulation of IRF1 during exit from naive pluripotency confers viral protection
Source: EMBO Rep. 2022 Jul 19;23(9):e55375. doi: 10.15252/embr.202255375 (PMC9442322; doi:10.15252/embr.202255375)
Supplement: Supplementary file 1 — Appendix [file EMBR-23-e55375-s007.pdf]

Transient upregulation of IRF1 during the exit from naive pluripotency confers viral protection

Merrit Romeike[1,3], Stephanie Spach[1], Marie Huber[1], Songjie Feng[1,3], Gintautas Vainorius[2,3], Ulrich Elling[2], Gjis A. Versteeg[1], Christa Buecker[1]*

[1]Max Perutz Labs Vienna, Vienna Biocenter (VBC), University of Vienna, Dr.Bohr-Gasse 9, 1030 Vienna, Austria
[2]Institute of Molecular Biotechnology of the Austrian Academy of Science (IMBA), Vienna Biocenter (VBC), Dr. Bohr-Gasse 3, 1030 Vienna, Austria
[3]Vienna Biocenter PhD Program, a Doctoral School of the University of Vienna and Medical University of Vienna, A-1030 Vienna, Austria
*corresponding author

Appendix

Table of Contents

Appendix Figure S1 2

Appendix Figure S2 3

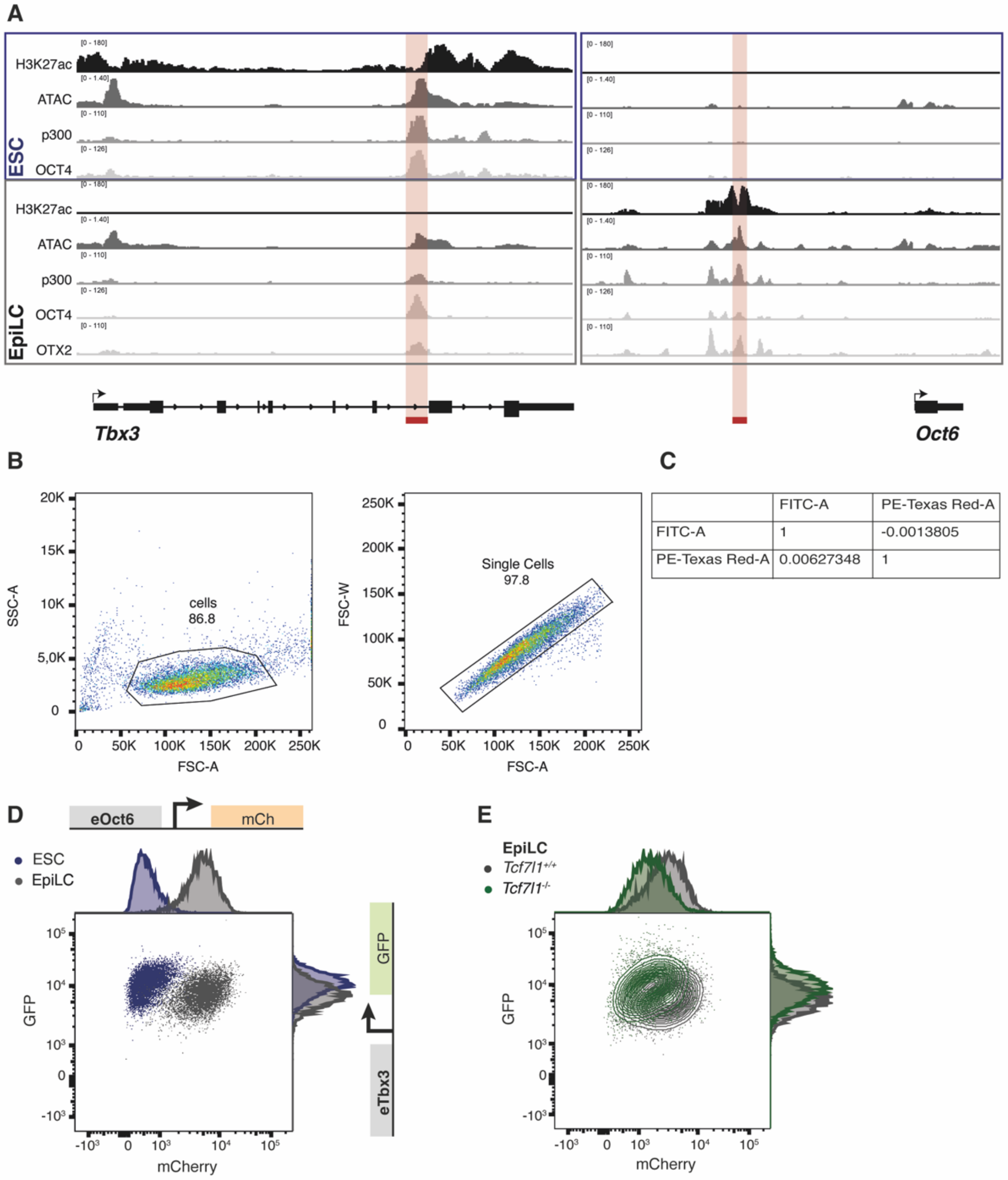

	FITC-A	PE-Texas Red-A
FITC-A	1	-0.0013805
PE-Texas Red-A	0.00627348	1

Appendix Figure S1 related to Figure 1

Appendix Figure S1 related to Figure 1

A Chromatin context of *Tbx3* and *Oct6* loci in ESC and EpiLC. Enhancer regions used as reporters are indicated by red boxes. ChIP data was generated by Buecker et al. 2014.

B Example gating strategy for FACS analysis of screening cell lines.

C Compensation matrix for the used fluorescent reporter constructs, eTbx3-GFP measured in FITC-A, eOtc6-mCherry measured in PE-Texas-Red channels.

D Representative flow cytometry profiles of reporter cell lines in ESC and differentiated to EpiLC conditions.

E Representative flow cytometry profiles of reporter cell lines in EpiLC condition, *Tcf7l1*$^{+/+}$ and *Tcf7l1*$^{-/-}$.

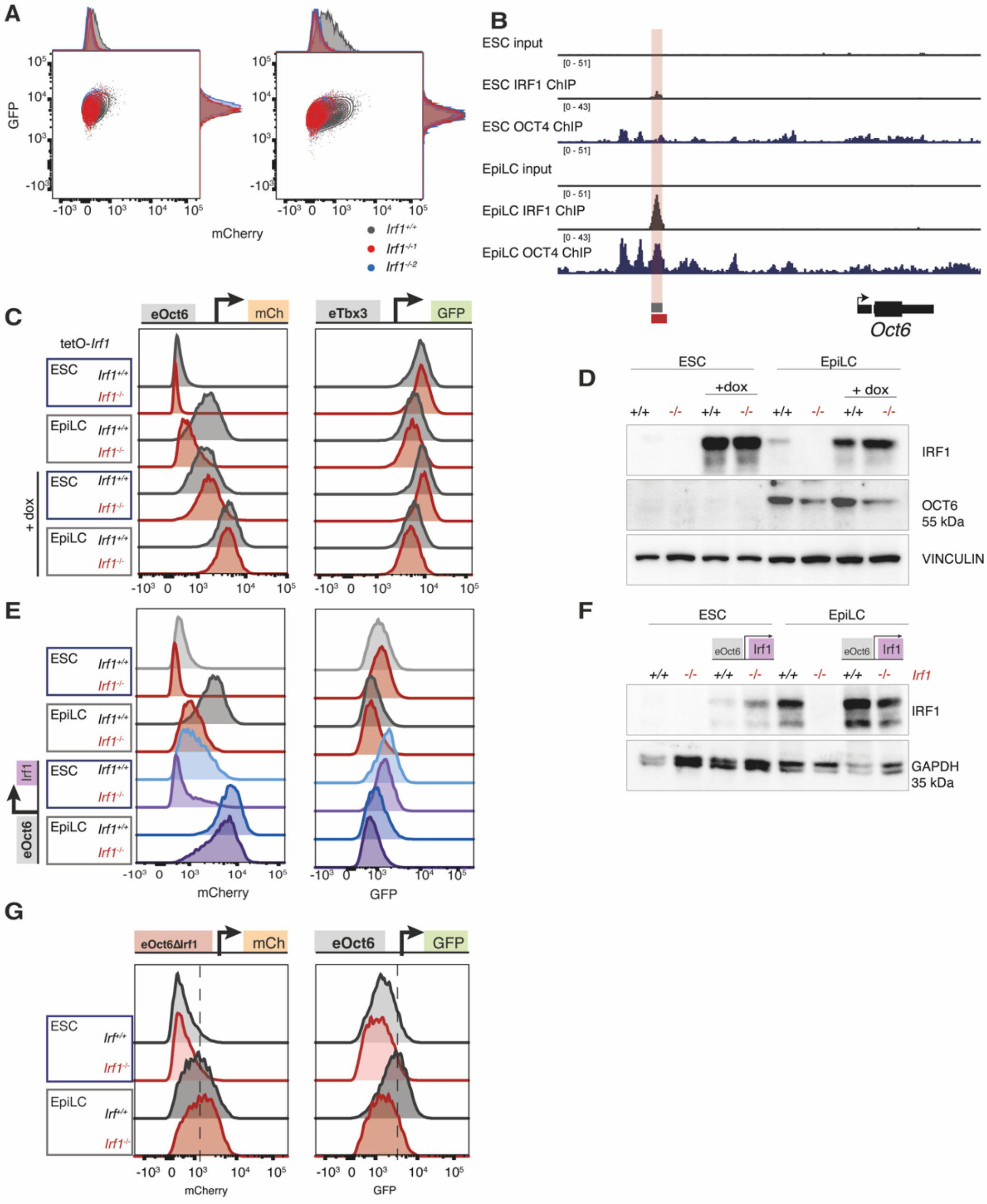

Appendix Figure S2 related to Figure 4

Appendix Figure S2 related to Figure 4

A Representative flow cytometry profiles of reporter cell lines in ESC and EpiLC conditions, *Irf1*$^{+/+}$ and *Irf1*$^{-/-}$. Shown are two independent cell lines.

B Chromatin context of *eOct6* locus, with a called IRF1 peak highlighted. IRF1 ChIP-seq tracks and OCT4 ChIP-seq tracks for ESC and EpiLCs are shown. The *eOct6* region used as reporter is marked with the lower red box. OCT4 ChIP-Seq data from Buecker et al., 2014.

C Representative flow cytometry profiles of reporter cell lines in ESC and EpiLC condition, *Irf1*$^{+/+}$ and *Irf1*$^{-/-}$ and doxycycline-induced IRF1 expression.

D Western Blot analysis of doxycycline-induced IRF1 expression in *Irf1*$^{+/+}$ and *Irf1*$^{-/-}$ background, in ESC and EpiLC cells, probed with antibodies against IRF1, OCT6 and GAPDH as loading control.

E Representative flow cytometry profiles of reporter cell lines in ESC and EpiLC condition, *Irf1*$^{+/+}$ and *Irf1*$^{-/-}$ and ectopic IRF1 expression driven by the *eOct6* enhancer.
F Western Blot analysis of ectopic IRF1 expression in Irf1$^{+/+}$ and Irf1$^{-/-}$ background, in ESC and EpiLC cells, probed with antibodies against IRF1 and GAPDH as loading control.
G Representative flow cytometry profiles of ESC and EpiLC cells, *Irf1*$^{+/+}$ and *Irf1*$^{-/-}$ with *eOct6ΔIrf* and *eOct6* controlling mCherry and GFP, respectively.

--- end of appendix --
