# Supplementary material for: Transient upregulation of IRF1 during exit from naive pluripotency confers viral protection
Source: EMBO Rep. 2022 Jul 19;23(9):e55375. doi: 10.15252/embr.202255375 (PMC9442322; doi:10.15252/embr.202255375)
Supplement: Supplementary file 2 — Expanded View Figures PDF [file EMBR-23-e55375-s005.pdf]

# Expanded View Figures

A

| set | genes | guides |
|---|---|---|
| Transcription Factors | 845 | 3290 |
| Epigenetic Regulators | 548 | 2134 |
| Nuclear Genes Set 1 | 1994 | 7765 |
| Nuclear Genes Set 2 | 2072 | 8069 |
| DNA repair | 265 | 1437 |
| non-targeting control | | 86 |
| total | 2524 | 22781 |

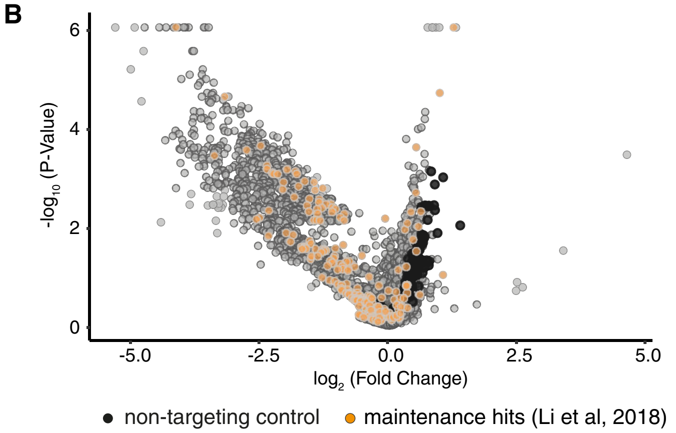


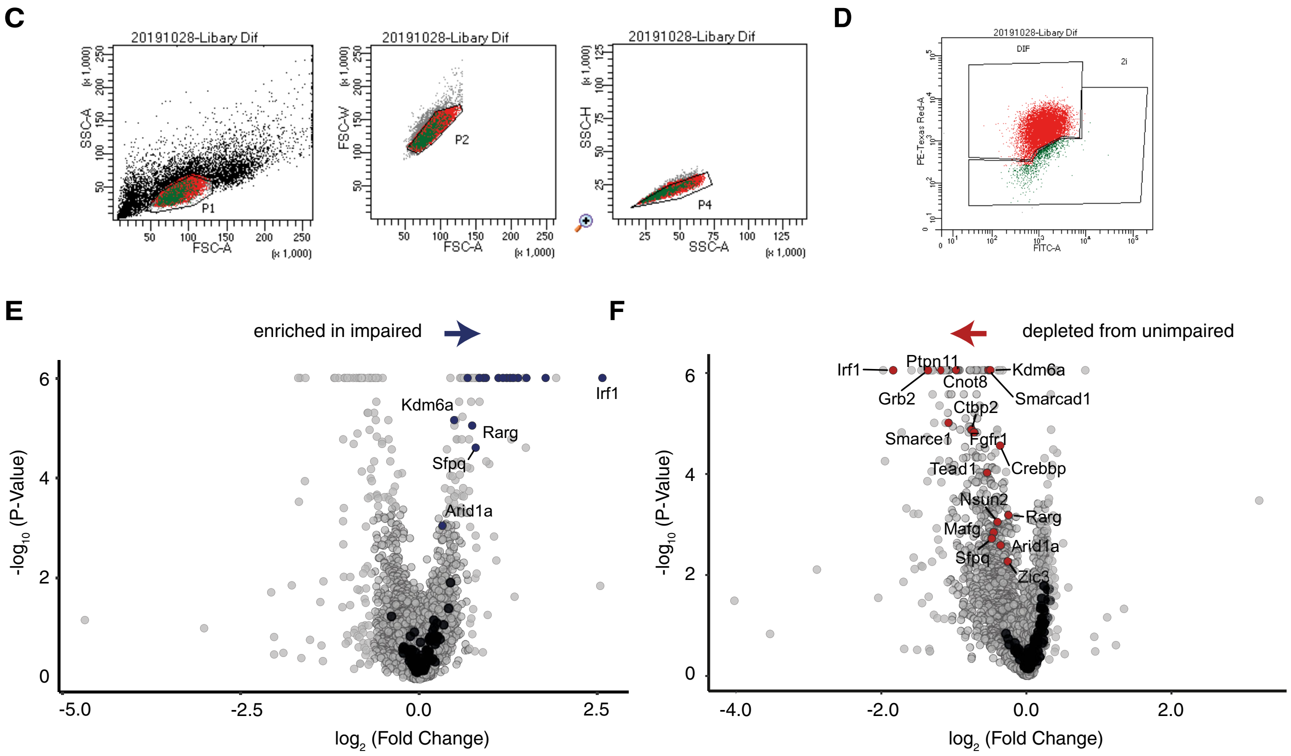


**Figure EV1. Screen strategy to identify factors required for activation of formative pluripotency.**

A Information about sgRNA library used in CRISPR-KO screen. Shown are number of targeted genes and unique guides used for each class of genes.

B As in Fig 1B, sgRNA representation in ESCs with CAS9 vs. ESCs without CAS9. Indicated are known ESC maintenance genes (Li *et al*, 2018).

C Example screening FACS gating strategy for CRISPR-KO screen for selection of single cells. Live cells were sorted according to FSC-A/SSC-A gates, followed by gating for FSC-A/FSC-W and SSC-A/SSC-H for individual cells.

D Example for gating strategy to sort impaired and unimpaired populations. Differentiation was scored by FITC-A/PE-TexasRed-A gating.

E As Fig 1C, sgRNA representation in EpiLC sorted as impaired vs. non-sorted EpiLC. Indicated in blue are factors identified as shared candidates (see Fig 1E).

F As Fig 1D, sgRNA representation in EpiLC sorted as unimpaired vs. non-sorted ESCs. Indicated in red are factors identified as shared candidates (see Fig 1E).

**Figure EV2. IRF1 expression in formative pluripotency is controlled by an EpiLC-specific enhancer.**

A Genotyping PCR of enhancer KO. Primers bind outside the edited site, producing smaller PCR products than on the control locus. Enhancer KO A and C were generated with one set of sgRNA, enhancer KO B and D with a second, different set.

B Examples for Sanger sequencing of PCR products, shown are enhancer KO B and D. Gapped alignment is indicated by dashed lines.

C Western blot analysis of *Irf1*$^{+/+}$, *Irf1*$^{-/-}$ and *Irf1* enhancer KO ESC and EpiLC, probed with antibodies against IRF1, formative marker OTX2 and VINCULIN as loading control.

D Western blot analysis of *Irf1*$^{+/+}$ and *Irf1* enhancer KO ESC and EpiLC with ruxolitinib treatment, probed with antibodies against phosphorylated STAT3, IRF1 and VINCULIN as loading control.

E, F RT-qPCR analysis of *Tbx3* (E, naive marker) and *Otx2* (F, formative marker) mRNA in *Irf1*$^{+/+}$ and *Irf1* enhancer KO in differentiation, treated with ruxolitinib. Fold change is normalized against *Rpl13a* housekeeping mRNA expression and is calculated against ESC for each indicated cell line. This is shown as baseline with the dashed line at fold change = 1. Shown are three biological replicates, datapoints from the same replicate are indicated with the same symbol. Note that each replicate contains two *Irf1*$^{+/+}$ samples. Black horizontal lines show the mean of the data.

G *Irf1* expression [FPKM] in murine embryos (Data ref: Boroviak *et al*, 2015b); $n = 3$ biological replicates.


© 2022 The Authors

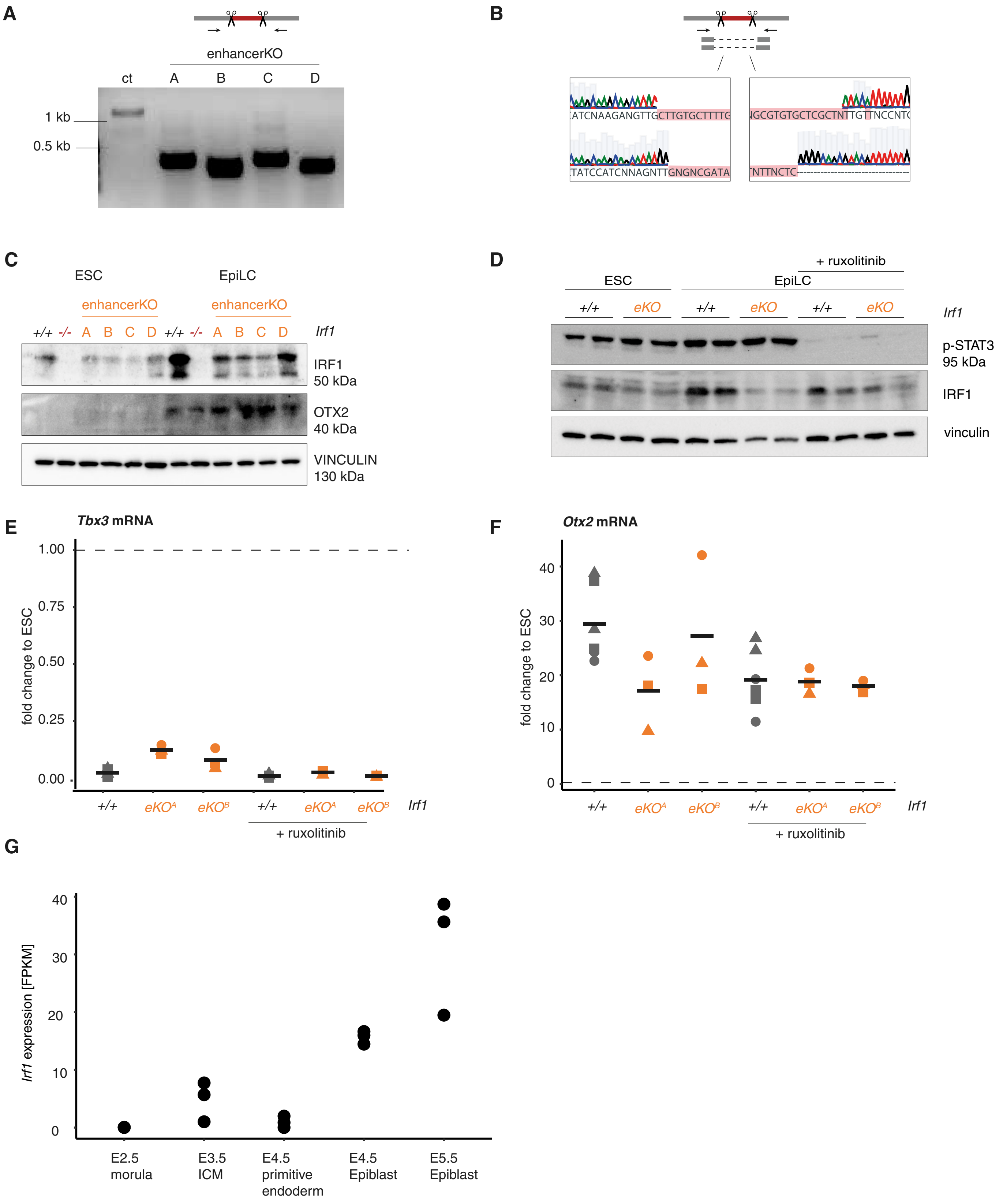
A
enhancerKO
ct
A
B
C
D
1 kb
0.5 kb
B
C
ESC
EpiLC
enhancerKO
enhancerKO
+/+ -/- A B C D +/+ -/- A B C D Irf1
IRF1 50 kDa
OTX2 40 kDa
VINCULIN 130 kDa
D
+ ruxolitinib
ESC
EpiLC
+/+ eKO +/+ eKO +/+ eKO Irf1
p-STAT3 95 kDa
IRF1
vinculin
E
Tbx3 mRNA
fold change to ESC
+/+ eKOA eKOB +/+ eKOA eKOB Irf1
+ ruxolitinib
F
Otx2 mRNA
fold change to ESC
+/+ eKOA eKOB +/+ eKOA eKOB Irf1
+ ruxolitinib
G
Irf1 expression [FPKM]
E2.5 morula
E3.5 ICM
E4.5 primitive endoderm
E4.5 Epiblast
E5.5 Epiblast

**Figure EV2.**

**Figure EV3. IRF1 is not a major regulator of pluripotency gene or repeat element expression.**

A Same Western blot as Fig 3A, long exposure and size marker of IRF1, VINCULIN is used as loading control. Overexposure is indicated with red signal.

B Expression changes in naive-associated genes (NAGs, Lackner *et al*, 2021) in $Irf1^{+/+}$ and $Irf1^{-/-}$ ESC and EpiLC based on QuantSeq RNA data. Data are shown as *z*-score. $n = 2$ biological replicates.

C Alkaline phosphatase staining of $Irf1^{+/+}$ and $Irf1^{-/-}$ in different cell seeding densities. Left panel: Cells were kept in 2i + LIF medium to control for seeding density, right panel: Cells were differentiated for 72 h before being placed back in 2i + LIF medium.

D MAplot of gene expression changes in EpiLC vs. ESC, ISGs are indicated. $n = 2$ biological replicates.

E MAplot of gene expression changes in EpiLC $Irf1^{-/-}$ vs. $Irf1^{+/+}$, ISGs are indicated. Differentially expressed (*P*-value < 0.05) Differentially expressed ISGs are plotted in Fig 3E. $n = 2$ biological replicates.

F Western blot analysis of dox-inducible SunTag-based IRF1 overexpression in ESCs, probed with antibodies against IRF1 and VINCULIN as loading control. Two different sgRNAs were tested, the sgRNA marked with an asterix (Irf1*) was used for RNA-sequencing.

G Dimension reduction (principal component analysis, PCA) plot of dox-inducible SunTag-based IRF1 overexpression in ESCs, based on QuantSeq RNA data. $n = 2$ biological replicates.

H Normalized counts for *Irf1* mRNA in dox-inducible SunTag-based IRF1 overexpression in ESCs, based on QuantSeq RNA data. *P*-value calculated by DESeq2. $n = 2$ biological replicates.

I Expression changes in dox-inducible SunTag-based IRF1 overexpression, left panel NAGS, right panel full set of ISGs. Data are shown as gene normalized *z*-score. Samples are color-coded as in Fig EV3G. $n = 2$ biological replicates.

J MAplot of TE family expression changes in EpiLC vs. ESC, adjusted *P*-values < 0.1 are indicated. $n = 2$ biological replicates.

K MAplot of TE family expression changes in EpiLC $Irf1^{-/-}$ vs. $Irf1^{+/+}$, adjusted *P*-values < 0.1 are indicated. $n = 2$ biological replicates.

 © 2022 The Authors

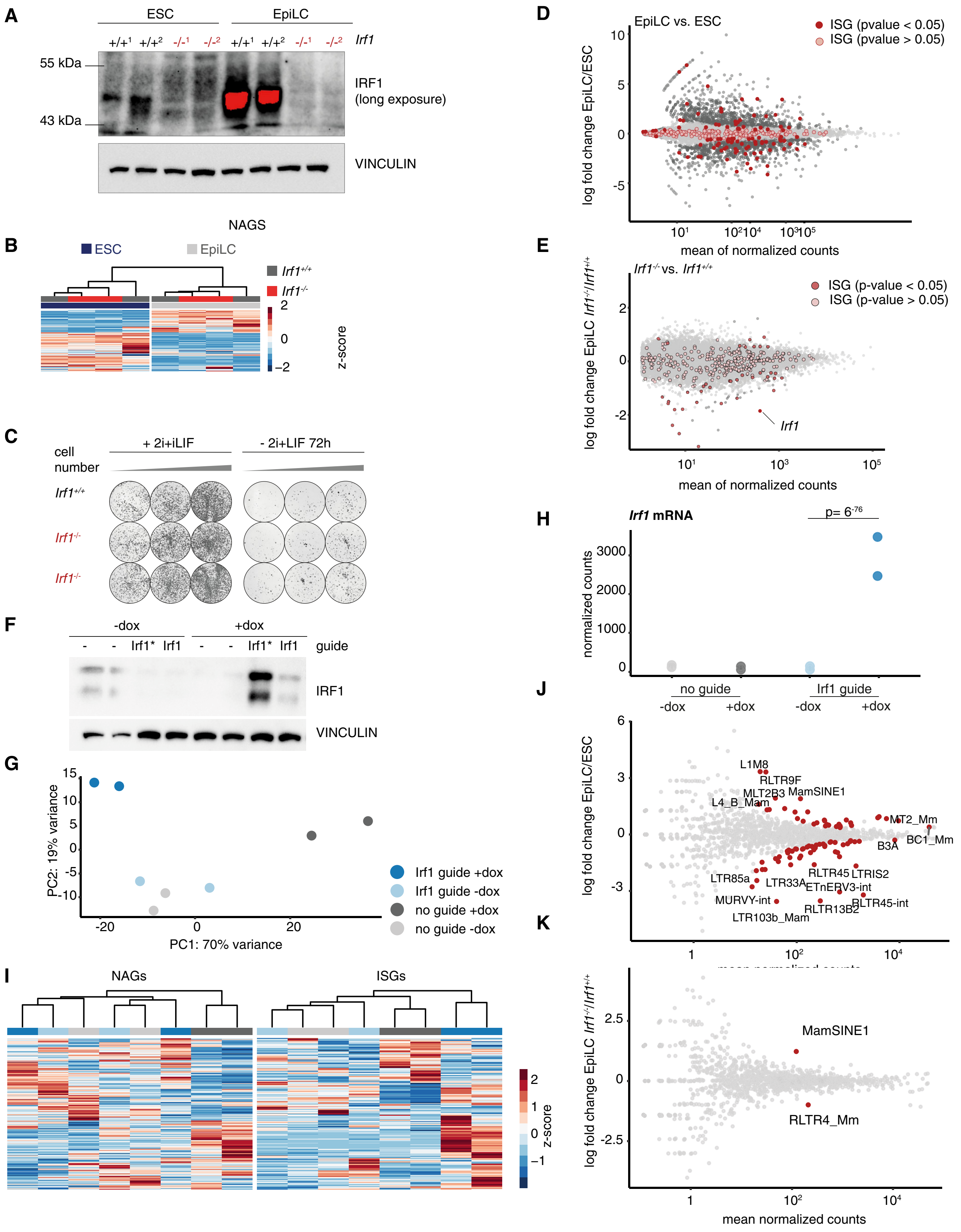
A
ESC
EpiLC
Irf1
55 kDa
43 kDa
IRF1
(long exposure)
VINCULIN
B
NAGS
ESC
EpiLC
z-score
C
cell number
+ 2i+iLIF
- 2i+LIF 72h
D
EpiLC vs. ESC
ISG (pvalue < 0.05)
ISG (pvalue > 0.05)
log fold change EpiLC/ESC
mean of normalized counts
E
ISG (p-value < 0.05)
ISG (p-value > 0.05)
Irf1
mean of normalized counts
F
-dox
+dox
guide
IRF1
VINCULIN
G
PC2: 19% variance
PC1: 70% variance
Irf1 guide +dox
Irf1 guide -dox
no guide +dox
no guide -dox
H
Irf1 mRNA
normalized counts
no guide
Irf1 guide
I
NAGs
ISGs
z-score
J
log fold change EpiLC/ESC
L1M8
RLTR9F
MLT2B3
MamSINE1
L4_B_Mam
MT2_Mm
BC1_Mm
B3A
LTR85a
LTR33A
RLTR45
LTRIS2
ETnERV3-int
MURVY-int
RLTR13B2
RLTR45-int
LTR103b_Mam
K
MamSINE1
RLTR4_Mm
mean normalized counts

**Figure EV3.**

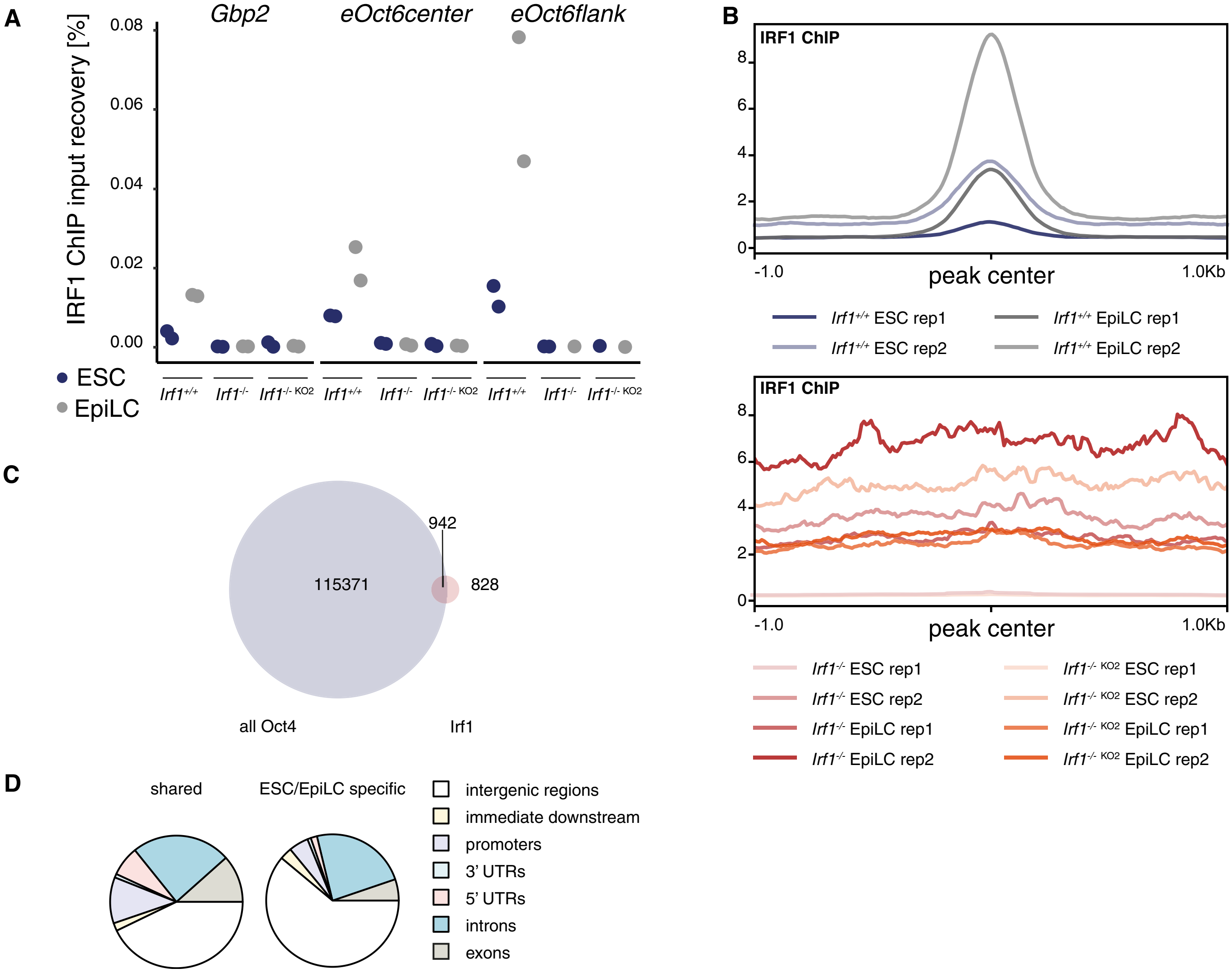


**Figure EV4. Chromatin binding of IRF1.**

A ChIP-qPCR analysis for the *Gbp2* promoter as known IRF1 binding site and two primer sets for the *eOct6* enhancer. Values are calculated as input recovery [%]. $n = 2$ biological replicates.

B Signal strength around all identified IRF1 binding sites for indicated conditions. Top panel: *Irf1*$^{+/+}$ ESC and EpiLCs, bottom panel: two independent *Irf1*$^{-/-}$ cell lines in ESC and EpiLC conditions. $n = 2$ biological replicates.

C Overlap of IRF1 binding sites and all OCT4 binding sites in ESCs and EpiLCs. (Data ref: Buecker *et al*, 2014b).

D Assigned chromatin regions of IRF1 binding sites shared between BMDMs and ESC/EpiLC (left) and IRF1 binding sites only detected in ESC/EpiLC (right).

 © 2022 The Authors

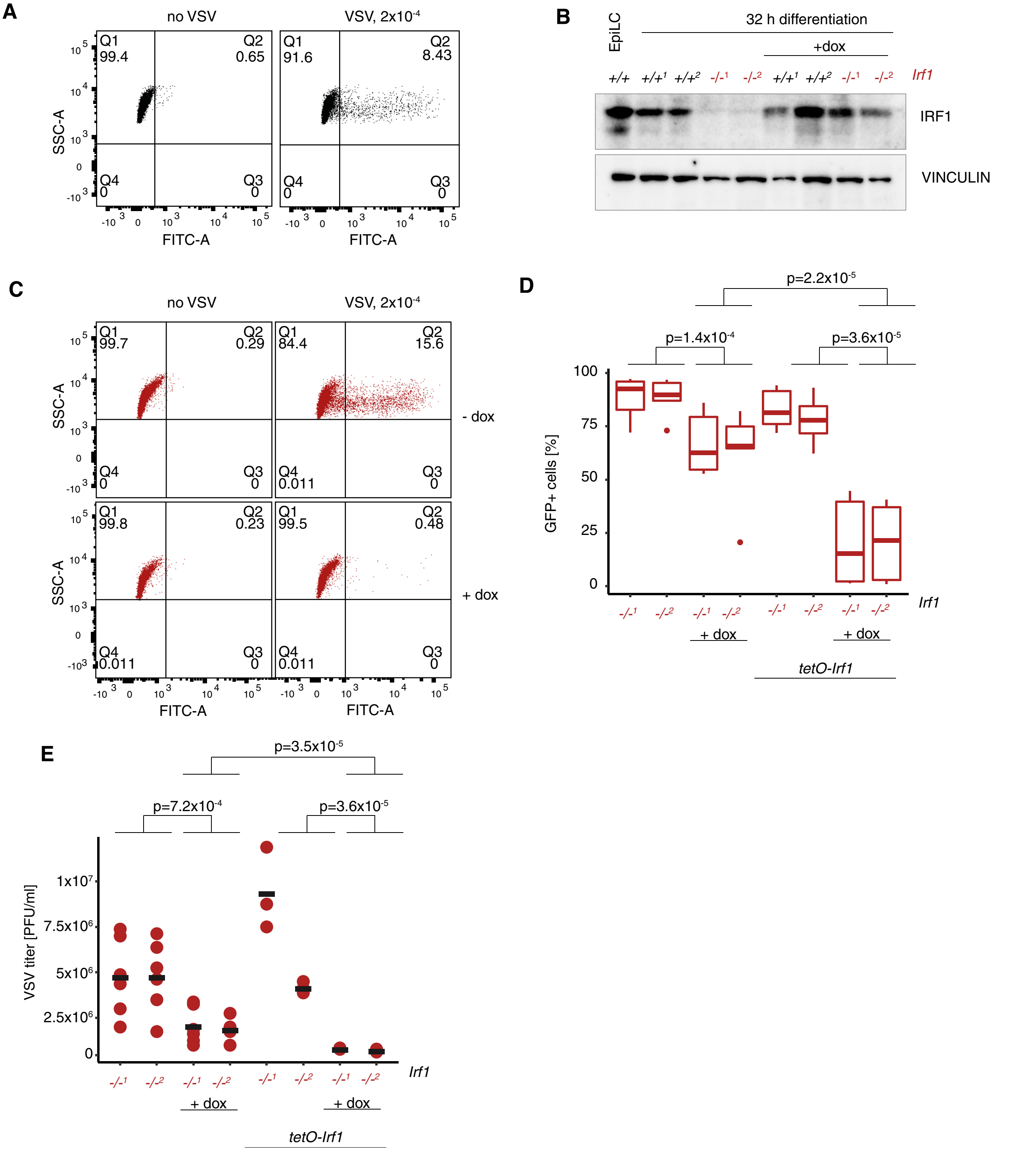
A
no VSV
VSV, 2x10-4
Q1 99.4
Q2 0.65
Q4 0
Q3 0
Q1 91.6
Q2 8.43
Q4 0
Q3 0
SSC-A
FITC-A
B
EpiLC
32 h differentiation
+dox
+/+ +/+1 +/+2 -/-1 -/-2 +/+1 +/+2 -/-1 -/-2 Irf1
IRF1
VINCULIN
C
no VSV
VSV, 2x10-4
Q1 99.7
Q2 0.29
Q4 0
Q3 0
Q1 84.4
Q2 15.6
Q4 0.011
Q3 0
- dox
Q1 99.8
Q2 0.23
Q4 0.011
Q3 0
Q1 99.5
Q2 0.48
Q4 0.011
Q3 0
+ dox
SSC-A
FITC-A
D
p=2.2x10-5
p=1.4x10-4
p=3.6x10-5
GFP+ cells [%]
-/-1 -/-2 -/-1 -/-2 -/-1 -/-2 -/-1 -/-2 Irf1
+ dox
+ dox
tetO-Irf1
E
p=3.5x10-5
p=7.2x10-4
p=3.6x10-5
VSV titer [PFU/ml]
1x10^7
7.5x10^6
5x10^6
2.5x10^6
0
-/-1 -/-2 -/-1 -/-2 -/-1 -/-2 -/-1 -/-2 Irf1
+ dox
+ dox
tetO-Irf1

**Figure EV5.**

◀

**Figure EV5. IRF1 in EpiLCs is required for protection against viral infection.**

A Representative FACS profiles of cells infected with GFP-VSV. Cells were scored according to FITC-A signal.

B Western blot analysis of IRF1 rescue with doxycycline-inducible *Irf1* construct. VINCULIN is used as loading control. All rescue samples were analyzed in two biological replicates.

C Representative FACS profiles of IRF1 doxycycline-inducible overexpression in cells infected with GFP-VSV, without and with doxycycline treatment. Cells were scored according to FITC-A signal.

D Quantification of $GFP^+$ cells after GFP-VSV infection (higher virus concentration, see Methods), $Irf1^{-/-}$ and doxycycline-inducible overexpression in $Irf1^{-/-}$ cells, without and with doxycycline induction. *P*-values were calculated per Wilcoxon test. $n = 6$ biological replicates. The central band shows the median, the box $25^{th}$ and $75^{th}$ percentiles, whiskers show 1.5*IGR (interquartile range).

E Viral titer as determined by plaque formation assays, $Irf1^{-/-}$ and IRF1 doxycycline-inducible overexpression in $Irf1^{-/-}$ cells, without and with doxycycline induction, as in panel (D). *P*-values were calculated per Wilcoxon test. $n = 3$ biological replicates for rescue, $n = 6$ for non-rescue conditions. Black horizontal lines show the mean of the data.

 © 2022 The Authors
