# Supplementary material for: Transient upregulation of IRF1 during exit from naive pluripotency confers viral protection
Source: EMBO Rep. 2022 Jul 19;23(9):e55375. doi: 10.15252/embr.202255375 (PMC9442322; doi:10.15252/embr.202255375)
Supplement: Supplementary file 11 [file EMBR-23-e55375-s011.pdf]

EMBO reports

Transient upregulation of IRF1 during exit from naive pluripotency confers viral protection

Merrit Romeike, Stephanie Spach, Marie Huber, Songjie Feng, Gintautas Vainorius, Ulrich Elling, Gijs Versteeg, and Christa Buecker

DOI: 10.15252/embr.202255375

Corresponding author(s): Christa Buecker (christa.buecker@univie.ac.at)

Review Timeline:

Transfer from Review Commons:	9th May 22
Editorial Decision:	30th May 22
Revision Received:	14th Jun 22
Accepted:	23rd Jun 22

Editor: Achim Breiling

Transaction Report: This manuscript was transferred to EMBO Reports following peer review at Review Commons.

(Note: With the exception of the correction of typographical or spelling errors that could be a source of ambiguity, letters and reports are not edited. Depending on transfer agreements, referee reports obtained elsewhere may or may not be included in this compilation. Referee reports are anonymous unless the Referee chooses to sign their reports.)

Review #1 -

1. Evidence, reproducibility and clarity:

Evidence, reproducibility and clarity (Required)

Summary

The paper by Romeike M et al describes the role of Irf1 during naïve to formative transition. They firstly identified the Irf1 as a key gene to activate formative specific enhancer of Oct6. The Irf1 KO ES cells failed to up-regulate the reporter however, the data showed here support that Irf1KO ES cells do not have a clear phenotype in the differentiation to the formative state. The authors also presented gain of function of Irf1 to induce ISGs in pluripotent stem cell context. The work was done properly and the conclusions from their results is reasonable. I have some suggestions to improve the manuscript.

Major Comments

1. It is unclear what is the expression pattern of Irf1 in vitro and in vivo. Fig. S3.1 C, D is not sufficient. Authors should show the expression pattern of mouse embryo from E3.5 to E6.5 to provide the background of the expression pattern during phase transitions, which is publicly available from a few previous studies. The human data in D did not state what type of the cells in each stage were examined. Authors should make this point clear. Irf1 is not expressed in naïve mES cells by western blot, but does IRF1 in human embryo express from naïve state? Also not detected by the western blot, there are some extent of Irf1 expression in naïve mouse ES cells? Fig 3C shows that the same Irf1 biding site can be ChIP in ES cells, but lesser than EpiLCs.
2. The rescue construct of Irf1 depends on the enhancer activity of Oct6, which depends on Irf1 itself (shown by the mCherry reporter in Fig. 3A). It is confusing to use this reporter system to rescue the KO because Irf1KO cannot use this enhancer. The leaky expression in the ES cell state enabled them to work as a rescue construct but dox inducible system authors used in the later experiments or simple dox inducible system is more appropriate to show.
3. Formative state of cells were recently captured as FS cells by Austin Smith's group. It might be worth trying to make FS cells from these Irf1 KO cells. The KO cells went into the formative state but it is unclear the gene and down stream ISGs play a key role for the maintenance.
4. Authors claim the expression of ISGs are intrinsic by citing the Wu et al paper. It is well known that Jak/Stat pathway is important for the naïve pluripotency but it is unclear in the post-implantation stage. It may be worth if authors could identify possible cytokines differentially expressed in formative state from the expression data, which induces Irf1 in formative specific manner.

Minor Comments

1. Page3. Is "mio" million?
2. It is interesting to show other outliner genes in Fig. 2B as well as informative to show some genes from 2E (17 genes) in panel C and D. Did authors identify Tcf7l1 in C or D?

3. The colours used in the most og the figures are very difficult to distinguish between samples. It would be helpful to use more distinct colours to see the different genotype etc to improve figures.
4. Irf1 western blots show consistent two bands. Please indicate/explain what they are. Also molecular size marker for the plot is missing.

2. Significance:

Significance (Required)

As authors stated, formative pluripotency is relatively new concept and it is not intensively examined yet. The results shown here identified Irf1 as one of the ISGs up-regulated in the formative state. The analysis support that the gene is not required for entering into formative phase, however, it is useful to report such a negative result to avoid the repeated experiments by the members of the stem cell society.

3. How much time do you estimate the authors will need to complete the suggested revisions:

Estimated time to Complete Revisions (Required)

(Decision Recommendation)

Between 1 and 3 months

Review #2

1. Evidence, reproducibility and clarity:

Evidence, reproducibility and clarity (Required)

In this paper, Romeike and colleagues provide a first molecular scenario linking gene regulatory networks associated with pluripotency and the interferon pathway. The study is robust and is well conducted. The conclusions are important and supported by the data. I particularly appreciated the acknowledgement of the limitations of the experimental setup used to identify regulators of formative pluripotency. While it is clear from the paper that Irf1 is not important by itself to enable the transition from naïve to formative pluripotency, I would suggest the authors to investigate the functional aspects associated with the increase in Irf1 upon exit from naïve pluripotency. A priori, one could think of functional relationships with antiviral or anti retrotransposon activation. It would be a great addition to this paper to explore these possibilities. This should be really taken as an advice to either improve the significance of this paper or as a follow up.

2. Significance:

Significance (Required)

The paper represents an important addition to our understanding of how the pluripotency networks connect with physiological properties of stem cells.

3. How much time do you estimate the authors will need to complete the suggested revisions:

Estimated time to Complete Revisions (Required)

(Decision Recommendation)

Cannot tell / Not applicable

Review #3

1. Evidence, reproducibility and clarity:

Evidence, reproducibility and clarity (Required)

Comments on Romeike et al.,:

In this manuscript, the authors screen for factors involved in the first step out of naïve pluripotency toward differentiation termed formative pluripotency. They identify the gene Irf1, which binds and upregulates Oct6, and genes involved in the innate immune response. They further identify the enhancer region of Irf1 that is controlled by the formative pluripotency gene regulatory network. Therefore, the authors functionally link the exit from naïve pluripotency to the noted changes in ISG expression.
The authors performed quite a lot of experiments and produced a large variety of data, and their findings are overall novel and interesting. The study is presented clearly, and it is a very interesting topic. However, several points should be addressed:

1. The authors ignore several key papers on the subject, such as Eggenberger et al., 2019 and Wang et al., 2013
2. The authors state " At the same time, stem cells are resistant against viral infection (Swartzendruber and Lehman 1975; Wolf and Goff 2009)." This is not an accurate interpretation of the papers and the sentence should be re-phrased and corrected.
3. The flow analysis data: raw data and compensated data files should be added as supplementary files.
4. Neomycin selection for 2 days is very short- explanation should be given in the Methods section
5. Better timeline of IRF1 expression should be drawn- it is not clear when the gene is expressed. Same for OCT6 - what is the correlation between their expressions in different developmental stages?
6. Figure 1B and figure S1B are identical, and one should be removed

- Eggenberger J, et al. (2019) Type I interferon response impairs differentiation potential of pluripotent stem cells. PNAS 116(4), pp. 1384-1393

- Wang R, et al. (2013) Mouse embryonic stem cells are deficient in type I interferon expression in response to viral infections and double-stranded RNA. J Biol Chem 288:15926-15936.

2. Significance:

Significance (Required)

the paper is of limited significance since the original attempt to define the formative pluripotency network was not achieved. the notion that IRF1 is responsible for the upregulation of other ISG at this stage is interesting and convincing, but since there are no effects on the pluripotency features, it is not groundbreaking.

A paper published in PNAS already described how overexpression of IRF7 resulted in dysregulation of many pluripotency and lineage-specific genes in both resting and differentiated iPSCs. The paper suggested that the IFN-I system is incompatible with maintaining a pluripotent state. the current manuscript doesn't provide this kind of significance.
the audience for this paper is hard-core stem cell people.
my expertise is epigenetics and stem cells.

3. How much time do you estimate the authors will need to complete the suggested revisions:

Estimated time to Complete Revisions (Required)

(Decision Recommendation)

Less than 1 month

Full Revision

Manuscript number: RC-2021-01188
Corresponding author(s): Christa Buecker

1. General Statements

We would like to thank all reviewers for their insightful comments, which helped us to significantly improve our work. We have now extensively revised the manuscript. Before addressing each of the individual points raised by the reviewers, we first discuss the major changes that have been incorporated in this transfer and their significance to our conclusions.

I. Viral protection of EpiLCs through IRF1

In our original submission we had identified *Irf1* in a CRISPR screen as a regulator of *Oct6* enhancer activity. *Irf1* is a well described member of so-called Interferon Stimulated Genes (ISGs) and we showed that *Irf1* is upregulated during the exit from naive pluripotency. Furthermore, we showed that the upregulation of *Irf1* is regulated through an enhancer that is activated during the exit from naive pluripotency. This enhancer is bound by transcription factors involved in the establishment of the formative pluripotent state, represented by OCT4 and OTX2.

Since IRF1 was shown to be involved in the innate immune response to viral infection, we speculated in the discussion that expression of IRF1 during the exit from naive pluripotency might be protective for the epiblast. In the revised version of the manuscript we have now included new data demonstrating that *Irf1* KO cells show increased susceptibility to viral infection compared to wild type cells (see Figure 5). Here, wild type and KO cells were infected with a GFP containing VSV virus. When IRF1 is not upregulated, either through deletion of *Irf1* itself or through deletion of the EpiLC specific enhancer, then a higher percentage of cells shows viral infection and the viral titers in the supernatant of the cells is strongly increased. Furthermore, overexpression of IRF1 in the *Irf1* KO cells reduces viral infection. Together these data demonstrate that IRF1 upregulation during early embryonic development protects the cells from viral infection.

II. Transient upregulation of *Irf1* during implantation of the blastocyst into the embryo

In our original submission we included data from Pijuan-Sala et al. 2019 showing that *Irf1* is also expressed in the developing embryo and in human embryos. Upon suggestion from Reviewer 1 and discussion with the editors, we expanded our data here and reanalyzed different RNA-seq datasets (single cell and bulk RN-Seq) for the expression of *Irf1* during the early embryonic development. First we analyzed the data from Kinoshita and colleagues (Kinoshita et al. 2020) from blastocsyt and the early pre- and post-implantation epiblast. Here, *Irf1* is expressed highest at day E5.0 and then expression is decreasing at day E5.5 and E6.0. Furthermore, we analyzed data from Boroviak et al to determine whether the high

expression of *Irf1* occurs already before implantation. Here highest expression of *Irf1* was observed at day E5.5, before that expression of *Irf1* in the epiblast was lower. Of note, the datasets are only partially comparable, E5.0 was not analyzed by Boroviak and colleagues. But together, these data suggest that *Irf1* expression occurs transiently during the implantation process, when the developing embryo makes first contact with the mother. Finally, we analyzed a human data set data from Mole et. al.: here human blastocyst were cultured *in vitro* up until 11 d.p.f. and single cells were analyzed at 5 d.p.f., 9 d.p.f. and 11 d.p.f.. *Irf1* is lowly expressed before the implantation occurs at 5 d.p.f., however, at 9 d.p.f. and 11 d.p.f. *Irf1* expression is upregulated in the developing human embryo, suggesting that *Irf1* upregulation is conserved between human and mouse. We included these data in the revised Figure 2 and Figure S3.

Together with our data on viral infection, we propose in the discussion that transient upregulation of *Irf1* during the implantation phase is a prophylactic measure to protect the embryo during this critical phase of development.

III. *Irf1* regulation through JAK/STAT

Irf1 is an ISG and therefore in many tissues under direct control of the JAK/STAT pathway. Pluripotency is in part governed through the LIF-STAT3 signaling axis. The JAK/STAT pathway is therefore of importance during early embryonic development. However, our model suggests that an EpiLC specific enhancer is mainly responsible for the upregulation of *Irf1*, and that thereby the formative pluripotency network is driving *Irf1* expression during early embryonic development. To exclude that JAK/STAT is involved in the upregulation of *Irf1*, we treated mESCs with ruxolitinib, a pan JAK Kinase inhibitor before and during the differentiation to EpiLCs. Here, Irf1 is still upregulated despite treatment with ruxolitinib. We included these data in our revised figure 2 C and Figure S3. Therefore we conclude that JAK/STAT during early embryonic development has only a minor effect on *Irf1* expression and that *Irf1* expression is driven largely by the formative specific network.

The new data incorporated into the revised manuscript lead to a reorganization and extension of the revised manuscript. The main conclusions were strengthened through additional data.

2. Point-by-point description of the revisions

<u>Reviewer #1</u>

We thank this reviewer for his/her thorough, constructive and helpful comments. Below we offer a point by point response to the individual points raised by the reviewer.

> *It is unclear what is the expression pattern of Irf1 in vitro and in vivo. Fig. S3.1 C, D is not sufficient. Authors should show the expression pattern of mouse embryo from E3.5 to E6.5 to provide the background of the expression pattern during phase transitions, which is publicly available from a few previous studies. The human data in D did not state what type of the cells in each stage were examined. Authors should make this point clear. Irf1 is not expressed in naïve mES cells by western*

> *blot, but does IRF1 in human embryo express from naïve state? Also not detected by the western blot, there are some extent of Irf1 expression in naïve mouse ES cells? Fig 3C shows that the same Irf1 biding site can be ChIP in ES cells, but lesser than EpiLCs.*

We thank the reviewer for the comments. We have now taken advantage of publicly available datasets from Boroviak et al (2015), Kinoshita et al (2020) and the web browser from Mittnenzweig et al (2021). All of these datasets stress that *Irf1* expression coincides with the short time window of implantation, as discussed above.
Similarly, we analyzed a human dataset from Mole et al. (2021). In this study, the authors integrated data from human pre-implantation ICM at 5 d.p.f. with data derived from post-implantation epiblast at 9 and 11 d.p.f. Strikingly, *Irf1* upregulation occurs also around the same time point when implantation occurs in human. These data confirm that *Irf1* upregulation is not an in-vitro effect of our culture conditions. Furthermore, it suggests that the role of *Irf1* in embryo development could be evolutionary conserved. We have included these data now in Figures 2D, 2E, S3G.

Irf1 is expressed lowly in naive conditions but gets upregulated strongly during differentiation. We now included a longer exposure time for the IRF1 Western Blot (Figure S4A), which shows low expression levels in naive conditions. These low expression levels and also binding sites identified by ChIP-seq in wildtype ESCs are distinct from *Irf1* KO samples, where all signals and enrichment is gone. *Irf1* expression is not completely off in naive conditions, a low level of protein can be seen under naive conditions. When we enrich the expressed protein using ChIP-assays, IRF1 in ESCs binds to its regular targets that are also identified during differentiation. In ESCs expression is low in all cells, as can be observed in the IF staining shown in Figure 3B. In sum, we conclude that *Irf1* is upregulated during the differentiation from ESC to EpiLCs.

> *The rescue construct of Irf1 depends on the enhancer activity of Oct6, which depends on Irf1 itself (shown by the mCherry reporter in Fig. 3A). It is confusing to use this reporter system to rescue the KO because Irf1KO cannot use this enhancer. The leaky expression in the ES cell state enabled them to work as a rescue construct but dox inducible system authors used in the later experiments or simple dox inducible system is more appropriate to show.*

We agree with the reviewers' concerns and now included an inducible IRF1 expression construct based on the well described doxycycline induction. We used this system in IRF1 KO cell lines in the dual reporter background used for screening to overexpress IRF1 (Fig S6C,D). Strikingly, addition of doxycycline under naive culturing conditions already drives expression of the *Oct6* enhancer reporter, without influencing the *Tbx3* enhancer reporter. This vast *Oct6* enhancer activation is not reflected in expression of OCT6 protein (Fig S6D), potentially because under naive conditions the chromatin state of the *Oct6* promoter region is not accessible yet. This also stresses that IRF1 is one of several factors required for *Oct6* expression, which is also reflected in reduced, but not abolished expression levels of OCT6 in *Irf1* KO.

Nevertheless, we still regard the *Oct6* enhancer driven expression of IRF1 as a tool to couple differentiation onset and *Irf1* expression, and therefore included this experiment as a supplementary figure (Fig S6E.F). Even in the absence of IRF1, the *Oct6* enhancer can be activated to a diminished extend, and rescue of IRF1 expression is possible in the *Irf1* KO cells. Both datasets complement each other well: the dox inducible system leads to strong overexpression while the Oct6 enhancer based system leads to almost endogenous expression levels of Irf1.

> *3. Formative state of cells were recently captured as FS cells by Austin Smith's group. It might be worth trying to make FS cells from these Irf1 KO cells. The KO cells went into the formative state but it is unclear the gene and down stream ISGs play a key role for the maintenance.*

We thank the reviewer for this suggestion. As we described above, re-analysis of data from Austin Smith lab together with the study performed by Boroviak et al showed that *Irf1* is transiently upregulated with the highest expression occurring around E5.0, followed by subsequent downregulation. Interestingly, we can capture this expression using our transient differentiation system from ESCs into EpiLCs, where *Irf1* is upregulated within 48h of differentiation.

We checked a small single cell RNA-seq (SMARTseq2) of cells collected at 0, 12, 24 and 48 h of differentiation we have published previously (Thomas et al, 2021). Also here a rapid upregulation of *Irf1* is detectable:

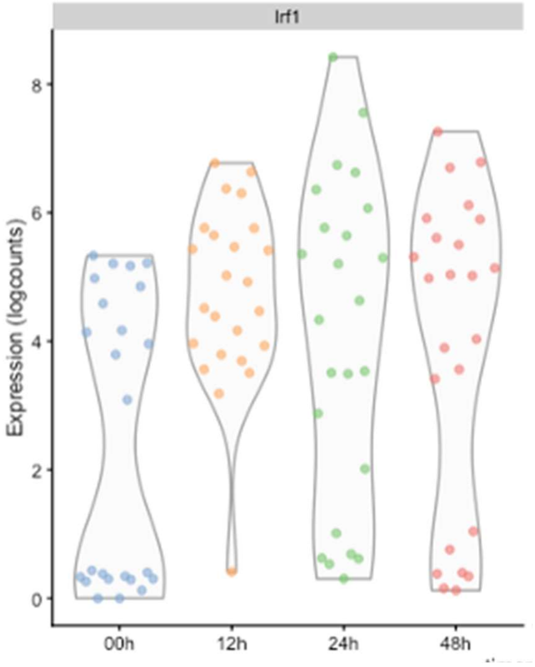

Figure 1: *Irf1* expression levels in single cells during differentiation using SMART-Seq

However, when we analyzed publicly available RNA-Seq from formative stem cells first reported by the Smith group, *Irf1* is not expressed highly (see Figure 2 below, X5cdr1 and X5cdr2 are two different formative stem cell lines derived by the Smith group). Therefore, the formative stem cell state might reflect a slightly later stage in development compared to the transient nature of the EpiLC differentiation. Indeed, formative stem cells seem to reflect the mouse embryo between E5.5 and E6.0 (Kinoshita et al. 2020). At these time points *Irf1* is already downregulated again in the embryo, once a stem cell state is captured and stabilized, the downregulation might be complete and *Irf1* is repressed. Why and how *Irf1* is downregulated will be an important question in the future albeit out of the scope for this manuscript. Therefore we did not include these data in the current manuscript.

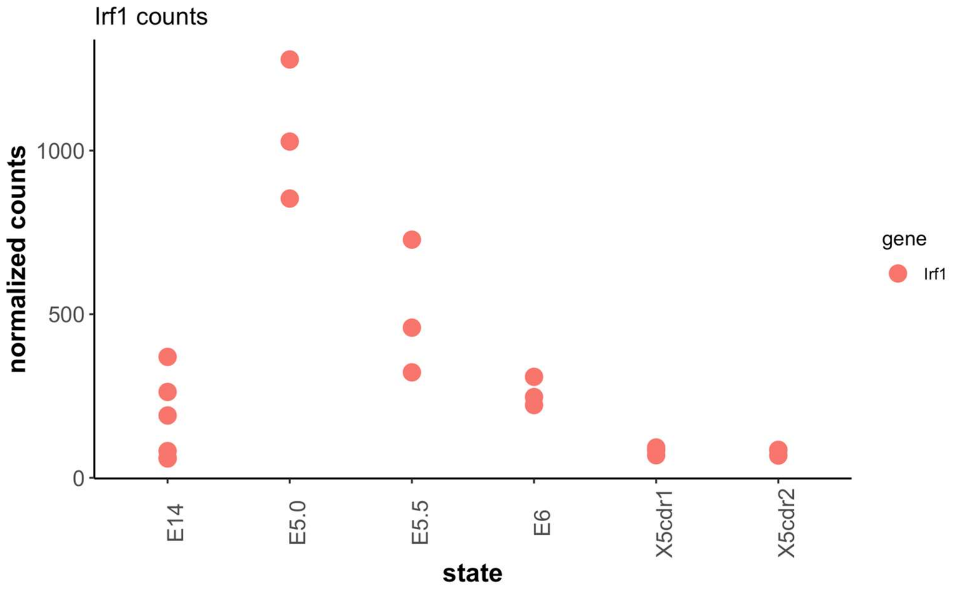

Figure 2: *Irf1* expression in early murine embryogenesis and in formative pluripotent stem cells

> *Authors claim the expression of ISGs are intrinsic by citing the Wu et al paper. It is well known that Jak/Stat pathway is important for the naïve pluripotency but it is unclear in the post-implantation stage. It may be worth if authors could identify possible cytokines differentially expressed in formative state from the expression data, which induces Irf1 in formative specific manner.*

We thank the reviewer for this suggestion. To address this, we first analyzed the contribution of JAK/STAT signaling to test whether this signaling pathway contributes to the expression of *Irf1* in formative pluripotency. We treated cells at the onset of differentiation with the Jak1/2 inhibitor ruxolitinib. The treatment was efficient as confirmed by drastic reduction of phospho-STAT3 levels. We compared the effect of JAK inhibition to the decrease of *Irf1* expression levels in *Irf1* enhancer KOs. JAK inhibition weakly decreases *Irf1* expression, but this decrease is not statistically significant while enhancer deletion leads to clear and statistically significant *Irf1* reduction.

We agree with the reviewer that it would be of interest to identify the cytokines that drive the expression of *Irf1* in a formative manner. However, this would require that we can disentangle differentiation and *Irf1* induction as two separate events: one cytokine that drives differentiation and a different one that drives *Irf1* upregulation. However, our data suggest a direct connection between differentiation and *Irf1* upregulation: the transcription factors that drive the entrance into formative pluripotency also activate *Irf1* expression. In our ChIP-Seq datasets we identified peaks of OCT4 and OTX2 at the enhancer of *Irf1* in the formative state. When we deleted this enhancer, *Irf1* expression is lower during differentiation, indicating that this enhancer is one of the main drivers of *Irf1* activation in formative pluripotency.
The increase of IRF1 expression in formative pluripotency is therefore caused by a combination of JAK/STAT Signaling and direct regulation through the formative pluripotency gene regulatory network through activation of an *Irf1* enhancer.

> *Minor Comments*
> *Page3. Is "mio" million?*

Yes, we changed the text to clarify this.

> *It is interesting to show other outliner genes in Fig. 2B as well as informative to show some genes from 2E (17 genes) in panel C and D. Did authors identify Tcf7l1 in C or D?*

We have labeled additional genes with known involvement in maintenance of naive pluripotency in Fig 1B. We now also included plots showing behavior of the 17 hits under each condition as supplement Figures S2 E+F. In addition to the raw sequencing data, we will also provide results tables from MAGeCK for each comparison, which allows convenient reanalysis of genes of interest.
Tcf7l1 was significantly depleted from the unimpaired population (p-value 0.004), but failed to pass the more stringent FDR cutoff (FDR 0.18). *Tcf7l1* did not show enrichment in the impaired population. The screens are not driven to saturation, therefore the absence of a phenotype for a given gene in the screen does not mean the gene is not involved in the faithful establishment of formative pluripotency.

> *The colours used in the most of the figures are very difficult to distinguish between samples. It would be helpful to use more distinct colours to see the different genotype etc to improve figures.*

We have adjusted the color schemes and hope that the differences between samples becomes clearer.

> *Irf1 western blots show consistent two bands. Please indicate/explain what they are. Also molecular size marker for the plot is missing.*

We have now included longer exposure times and size markers in Figure S4A and indicated molecular weights for all proteins blotted against.
For the double band, we see that the intensity of the double bands is changing depending on the lysis conditions in the SDS-PAGE sample preparation (see Figure 3 below). When we include phosphatase

inhibitors (PhosSTOP, Roche), the lower band is stabilized. IRF1 is phosphorylated, but phosphorylation would not be reflected in a lower molecular weight form as observed here (Garvin et al. 2019, doi: 10.1093/nar/gkz163**)**. We therefore cannot conclusively determine what the bands are, but both bands are specific and are not present in *Irf1* KOs. We have included a note on these observations in the methods section.

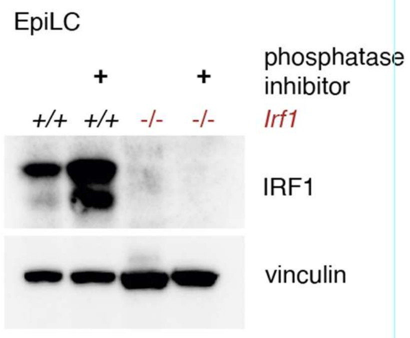

Figure 3: Phophatase inhibitor treatment of EpiLC protein lysates

Reviewer #1 (Significance (Required)):

As authors stated, formative pluripotency is relatively new concept and it is not intensively examined yet. The results shown here identified Irf1 as one of the ISGs up-regulated in the formative state. The analysis support that the gene is not required for entering into formative phase, however, it is useful to report such a negative result to avoid the repeated experiments by the members of the stem cell society.

While IRF1 is not required for the establishment of the formative state, we now show in the revised version that loss of IRF1 leaves to higher susceptibility through viral infection. Therefore, this is an unexpected, but not a negative result.

Full Revision

Reviewer #2

In this paper, Romeike and colleagues provide a first molecular scenario linking gene regulatory networks associated with pluripotency and the interferon pathway. The study is robust and is well conducted. The conclusions are important and supported by the data. I particularly appreciated the acknowledgement of the limitations of the experimental setup used to identify regulators of formative pluripotency. While it is clear from the paper that Irf1 is not important by itself to enable the transition from naïve to formative pluripotency, I would suggest the authors to investigate the functional aspects associated with the increase in Irf1 upon exit from naïve pluripotency. A priori, one could think of functional relationships with antiviral or anti retrotransposon activation. It would be a great addition to this paper to explore these possibilities. This should be really taken as an advice to either improve the significance of this paper or as a follow up.

We thank the reviewer for the favorable assessment of the manuscript. We now included an analysis of expression of transposable elements in the RNAseq data (see Figure S4J,K). Interestingly, differentiation has an effect on expression of several families of TEs, however, *Irf1* KO EpiLCs only show two misregulated families. We therefore conclude that IRF1 is not a major regulator of TE activity.
We investigated whether *Irf1* contributes to viral defense in the transition from naive to formative pluripotency. We reasoned that many genes upregulated by IRF1 are involved in viral protection and might therefore protect the cells from viral infection during the exit from naive pluripotency. For these experiments, naive cells were differentiated for 32 hours to allow for robust expression of IRF1 in wildtype controls, and then challenged with VSV-GFP for 16 hours. The infection rate was determined by FACS analysis. Strikingly, full IRF1 KO as well as KOs of the *Irf1* enhancer regulated by the formative pluripotency network showed a strong increase in infection rates. In addition, viral titers were higher in the different KO cell lines compared to control cells. Finally, ectopic expression of IRF1 lowered infection rates and viral titers. We therefore conclude that faithful IRF1 expression in formative pluripotency is indeed functionally relevant in defense against viral infections. We believe that this phenotype increases the significance of our manuscript.

Reviewer #2 (Significance (Required)):
The paper represents an important addition to our understanding of how the pluripotency networks connect with physiological properties of stem cells.

We thank the reviewer for this assessment.

Full Revision

Reviewer #3

Evidence, reproducibility and clarity (Required):
In this manuscript, the authors screen for factors involved in the first step out of naïve pluripotency toward differentiation termed formative pluripotency. They identify the gene Irf1, which binds and upregulates Oct6, and genes involved in the innate immune response. They further identify the enhancer region of Irf1 that is controlled by the formative pluripotency gene regulatory network. Therefore, the authors functionally link the exit from naïve pluripotency to the noted changes in ISG expression.
The authors performed quite a lot of experiments and produced a large variety of data, and their findings are overall novel and interesting. The study is presented clearly, and it is a very interesting topic.

We would like to thank the reviewer for their critical comments and have addressed each of the points raised below.

However, several points should be addressed:
1. The authors ignore several key papers on the subject, such as Eggenberger et al., 2019 and Wang et al., 2013
• Eggenberger J, et al. (2019) Type I interferon response impairs differentiation potential of pluripotent stem cells. PNAS 116(4), pp. 1384-1393

Eggenberger et al. use an elegant system to study differences in response to viruses between iPSCs and fibroblasts. In their study they show that fibroblast, but not iPSCs, respond with upregulation of IRF7 to IFNB. Forced expression of an active form of IRF7 in iPSCs elicits an antiviral response, but is not compatible with maintenance of pluripotency.

We tested expression levels of all IRF family members in naive and formative cells (see Figure 4 below). *Irf1* is the only IRF family member which is upregulated in differentiation; *Irf3*, *Irf6* and *Irf9* show robust expression, but are not changing between cell states. *Irf2*, *Irf4*, *Irf5* and *Irf8* are weakly expressed, *Irf7* is not expressed. *Irf7* is therefore not involved in physiological protection of stem cells against viral infection, even though it can take over that role if expression is extrinsically regulated.

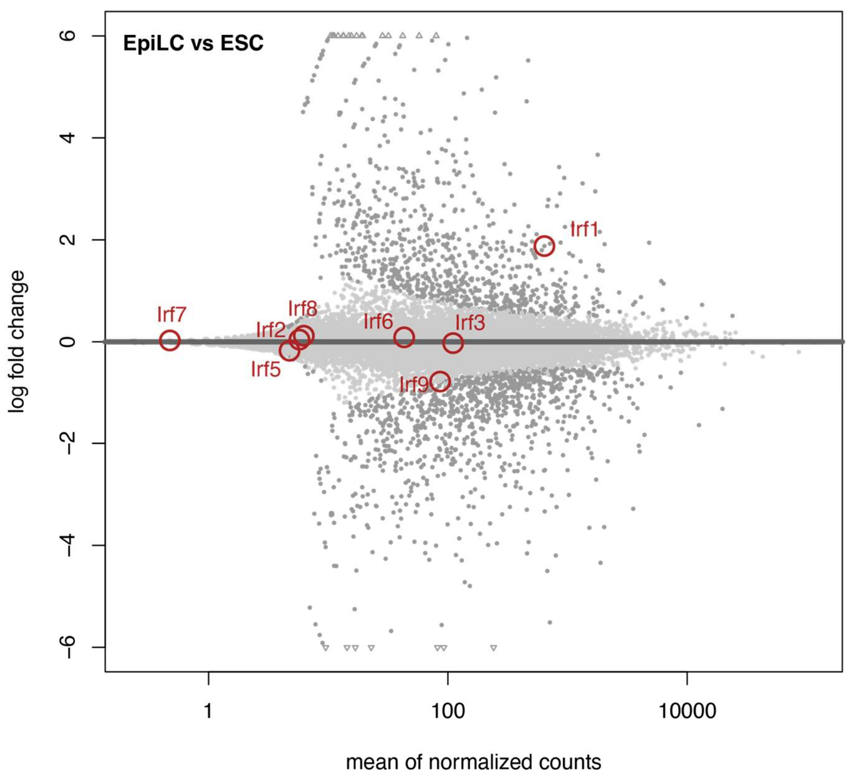

Figure 4: Expression of IRF family members in ESCs and EpiLCs

In contrast, IRF1 expression is intrinsically regulated, stressing its involvement in intrinsic viral protection. IRF1 expression is compatible even with naive pluripotency, as we do not observe misregulation of naive pluripotency factors or cell death under IRF1 overexpression in naive ESCs.

We have included a reference to Eggenberger et al in the context of ISG expression.

- *Wang R, et al. (2013) Mouse embryonic stem cells are deficient in type I interferon expression in response to viral infections and double-stranded RNA. J Biol Chem 288:15926-15936.*

Wang et al. demonstrate that mESCs are deficient in type I IFN expression, in part due to a lack of TLR3 surface receptors. However, Wu et al showed that different stem cell systems show an intrinsic expression of ISGs even in the absence of a functional type I interferon response. In our manuscript we show that IRF1 is a key driver of a subset of ISGs in the absence of viral infection and that *Irf1* expression is regulated through a cell state specific enhancer and not through the canonical interferon response through the JAK/STAT pathway. This prophylactically activated state of the defense system could be especially important in light of the findings of Wang et al. and we have included the reference in the introduction.

2. The authors state " At the same time, stem cells are resistant against viral infection (Swartzendruber and Lehman 1975; Wolf and Goff 2009)." This is not an accurate interpretation of the papers and the sentence should be re-phrased and corrected.

We understand that is paragraph was not stated clearly and have rephrased it to:

“Pluripotent and multipotent stem cells intrinsically express subsets of genes which are referred to as interferon stimulated genes (ISGs) (Wu et al. 2018). The subsets of expressed ISGs are distinct between different stem cell types and change in differentiation (Wu et al. 2018). How these ISGs are regulated is unknown, as mouse embryonic stem cells for example do not respond to interferon (IFN) stimulation (Burke, Graham, and Lehman 1978; Harada et al. 1990; Chen et al. 2020; Wang et al. 2013). Strikingly, pluripotent stem cells are more resistant against viral infection compared to their differentiated descendants (Swartzendruber and Lehman 1975; Wolf and Goff 2009) and this is at least in part due to ISG expression (Wu et al. 2018). However, whether ISGs and pluripotency gene expression are functionally connected is so far not known.”

3. The flow analysis data: raw data and compensated data files should be added as supplementary files.

We have included examples for gating strategies and a compensation matrix of the used fluorophores in the supplements (Figure S1B, S1C, S2C, S2D)

4. Neomycin selection for 2 days is very short- explanation should be given in the Methods section

We are using G418 in 400 µg/ml concentration, which are very stringent selection conditions. In each replicate, a control with untransfected cells was included as a kill control. The kill controls showed consistently complete cell death after 2 days of selection.
We have stressed this point in the methods section.

5. Better timeline of IRF1 expression should be drawn- it is not clear when the gene is expressed. Same for OCT6 - what is the correlation between their expressions in different developmental stages?

We have reanalyzed the *in vivo* data published by Kinoshita et al (2020) (see also above and Figure 5) to determine *Irf1* and *Oct6* expression levels.

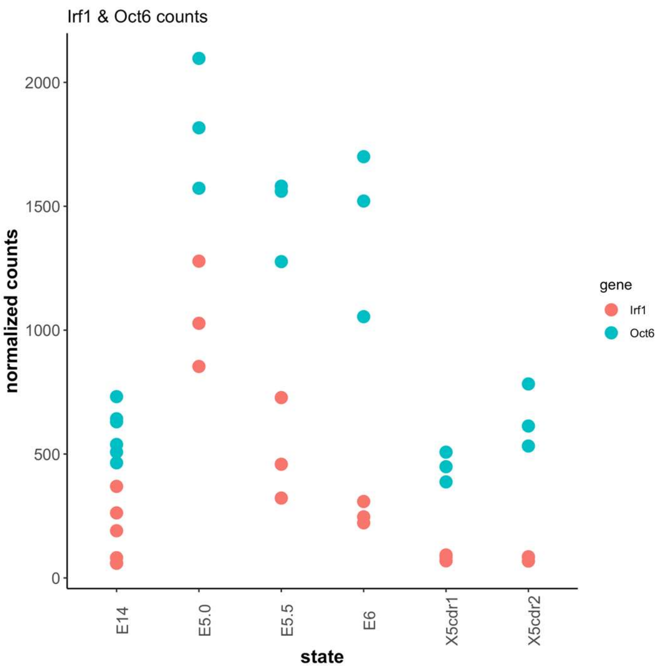

Figure 5: Expression of *Irf1* and *Oct6* in murine early embryogenesis based on Kinoshita et al.

Strikingly, both *Irf1* and *Oct6* show high expression at E5.0. *Irf1* expression drops at E5.5 and E6, while Oct6 expression remains high. This is in line with our results that *Irf1* is just one and not the sole regulator of *Oct6* expression.

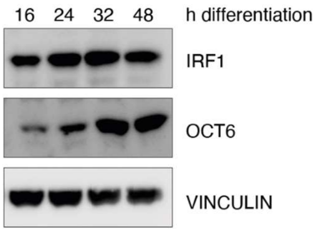

Figure 6: Protein levels of IRF1 and OCT6 in differentiation

We compared the induction kinetics of IRF1 and OCT6 in our differentiation system and performed a Western Blot time course spanning 16, 24, 32 and 48 hours of differentiation (see Figure 6). Here, IRF1 upregulation precedes OCT6 upregulation.

We did not include these data in the revised manuscript since we changed the focus from *Irf1*'s role in differentiation to its role in viral protection. Accordingly, we have toned down the statement that *Irf1* feeds back into the pluripotency network through regulation of *Oct6*.

6. Figure 1B and figure S1B are identical, and one should be removed

We performed our screen in two independently derived screening cell lines, to exclude clonal effects due to the several selection steps in cell line generation. We selected cell lines which were very consistent in their marker expression. Figure S1B was showing differentiation of the second cell line, FACSs plots are very similar, but not identical. We have now removed the original Figure S1B and included only one example of the screening cell line.

Reviewer #3 (Significance (Required)):

the paper is of limited significance since the original attempt to define the formative pluripotency network was not achieved. the notion that IRF1 is responsible for the upregulation of other ISG at this stage is interesting and convincing, but since there are no effects on the pluripotency features, it is not groundbreaking.
A paper published in PNAS already described how overexpression of IRF7 resulted in dysregulation of many pluripotency and lineage-specific genes in both resting and differentiated iPSCs. The paper suggested that the IFN-I system is incompatible with maintaining a pluripotent state. the current manuscript doesn't provide this kind of significance.
the audience for this paper is hard-core stem cell people.
my expertise is epigenetics and stem cells.

We do understand the reviewer's concerns, but we disagree with the statement that this study is of "limited significance" and only for "hard-core stem cell people".
While we identified IRF1 as a regulator of Oct6 enhancer activity and expression in the original screen, we demonstrate that the formative pluripotency network regulates IRF1 expression. Therefore, pluripotency and ISG expression are tightly connected and compatible. ISG expression in stem cells has been only described as "intrinsic" before, and here we show a direct regulation through the cell stage specific transcription factor network and not the classical interferon response.
In contrast, IRF7 is not expressed under physiological conditions as pointed out above, therefore the misregulation of pluripotency and lineage genes are specific for the forced expression. *Irf1* on the other hand is expressed in the *in vitro* system and during the implantation stage of the murine embryo. Even overexpression in mESC is compatible with pluripotency (see Figure S4I).
Furthermore, in the revised version of the manuscript we now show that IRF1 is important for protection of formative cells against viral infection. We therefore hypothesize that ISG expression, with IRF1 as a main regulator, in part protects formative stem cells independent from external signaling and viral recognition.

1st Editorial Decision **30th May 2022**

Dear Dr. Buecker,

Thank you for the submission of your revised manuscript to our editorial offices. I have now received the reports from the three referees that were asked to re-evaluate your study, you will find below. As you will see, the referees fully support publication of your work in EMBO reports.

Before we can proceed with formal acceptance, the manuscript needs formatting according to our journal style. Please carefully review the instructions that follow below.

PLEASE NOTE THAT upon resubmission revised manuscripts are subjected to an initial quality control prior to exposition to re-review. Upon failure in the initial quality control, the manuscripts are sent back to the authors, which may lead to delays. Frequent reasons for such a failure are the lack of the data availability section (please see below) and the presence of statistics based on n=2 (the authors are then asked to present scatter plots or provide more data points).

When submitting your final revised manuscript, we will require:

1) a .docx formatted version of the final manuscript text (including legends for main figures, EV figures and tables), but without the figures included. Figure legends should be compiled at the end of the manuscript text.

2) individual production quality figure files as .eps, .tif, .jpg (one file per figure), of main figures and EV figures. Please upload these as separate, individual files upon re-submission.

The Expanded View format, which will be displayed in the main HTML of the paper in a collapsible format, has replaced the Supplementary information. You can submit up to 5 images as Expanded View. Please follow the nomenclature Figure EV1, Figure EV2 etc. The figure legend for these should be included in the main manuscript document file in a section called Expanded View Figure Legends after the main Figure Legends section. Additional Supplementary material should be supplied as a single pdf file labeled Appendix. The Appendix should have page numbers and needs to include a table of content on the first page (with page numbers) and legends for all content. Please follow the nomenclature Appendix Figure Sx, Appendix Table Sx etc. throughout the text, and also label the figures and tables according to this nomenclature.

For more details, please refer to our guide to authors:
http://www.embopress.org/page/journal/14693178/authorguide#manuscriptpreparation

Please consult our guide for figure preparation:
http://wol-prod-cdn.literatumonline.com/pb-assets/embo-site/EMBOPress_Figure_Guidelines_061115-1561436025777.pdf

See also the guidelines for figure legend preparation:
https://www.embopress.org/page/journal/14693178/authorguide#figureformat

3) a complete author checklist, which you can download from our author guidelines (https://www.embopress.org/page/journal/14693178/authorguide). Please insert page numbers in the checklist to indicate where the requested information can be found in the manuscript. The completed author checklist will also be part of the RPF.

Please also follow our guidelines for the use of living organisms, and the respective reporting guidelines:
http://www.embopress.org/page/journal/14693178/authorguide#livingorganisms

4) that primary datasets produced in this study (e.g. RNA-seq, ChIP-seq, structural and array data) are deposited in an appropriate public database. If no primary datasets have been deposited, please also state this in a dedicated section (e.g. 'No primary datasets have been generated and deposited'), see below.

See also: http://embor.embopress.org/authorguide#datadeposition

Please remember to provide a reviewer password if the datasets are not yet public.

The accession numbers and database should be listed in a formal "Data Availability" section (placed after Materials & Methods) that follows the model below. This is now mandatory (like the COI statement). Please note that the Data Availability Section (DAS) is restricted to new primary data that are part of this study. This section is mandatory. As indicated above, if no primary datasets have been deposited, please state this in this section

Data availability

The datasets produced in this study are available in the following databases:

- RNA-Seq data: Gene Expression Omnibus GSE46843 (https://www.ncbi.nlm.nih.gov/geo/query/acc.cgi?acc=GSE46843)
- [data type]: [name of the resource] [accession number/identifier/doi] ([URL or identifiers.org/DATABASE:ACCESSION])

*** Note - All links should resolve to a page where the data can be accessed. ***

Moreover, I have these editorial requests:

6) We strongly encourage the publication of original source data with the aim of making primary data more accessible and transparent to the reader. The source data will be published in a separate source data file online along with the accepted manuscript and will be linked to the relevant figure. If you would like to use this opportunity, please submit the source data (for example scans of entire gels or blots, data points of graphs in an excel sheet, additional images, etc.) of your key experiments together with the revised manuscript. If you want to provide source data, please include size markers for scans of entire gels, label the scans with figure and panel number, and send one PDF file per figure.

7) Our journal encourages inclusion of *data citations in the reference list* to directly cite datasets that were re-used and obtained from public databases. Data citations in the article text are distinct from normal bibliographical citations and should directly link to the database records from which the data can be accessed. In the main text, data citations are formatted as follows: "Data ref: Smith et al, 2001" or "Data ref: NCBI Sequence Read Archive PRJNA342805, 2017". In the Reference list, data citations must be labeled with "[DATASET]". A data reference must provide the database name, accession number/identifiers and a resolvable link to the landing page from which the data can be accessed at the end of the reference. Further instructions are available at: http://www.embopress.org/page/journal/14693178/authorguide#referencesformat

8) Regarding data quantification and statistics, please make sure that the number "n" for how many independent experiments were performed, their nature (biological versus technical replicates), the bars and error bars (e.g. SEM, SD) and the test used to calculate p-values is indicated in the respective figure legends (also for potential EV figures and all those in the final Appendix). Please also check that all the p-values are explained in the legend, and that these fit to those shown in the figure. Please provide statistical testing where applicable. Please avoid the phrase 'independent experiment', but clearly state if these were biological or technical replicates. See also:
http://www.embopress.org/page/journal/14693178/authorguide#statisticalanalysis

9) Please also note our reference format:
http://www.embopress.org/page/journal/14693178/authorguide#referencesformat

10) For microscopic images, please add scale bars of similar style and thickness to all the microscopic images, using clearly visible black or white bars (depending on the background). Please place these in the lower right corner of the images. Please do not write on or near the bars in the image but define the size in the respective figure legend.

11) We updated our journal's competing interests policy in January 2022 and request authors to consider both actual and perceived competing interests. Please review the policy https://www.embopress.org/competing-interests and update your competing interests if necessary. Please name this section 'Disclosure and Competing Interests Statement'.

12) Please order the manuscript sections like this:
Title page - Abstract - Introduction - Results - Discussion - Materials and Methods - DAS - Acknowledgements - Author contributions - Disclosure and Competing Interests Statement - References - Figure legends - Expanded View Figure legends

13) I would suggest to upload the lists/tables in the methods section as 'Reagents and Tools table'. I have attached templates for that in word or excel format. Please upload the filled in table to the manuscript tracking system as a 'Reagent Table' file. The example below shows how the table will display in the published article and includes examples of the type of information that should be provided for the different categories of reagents and tools. Please list your reagents/tools using the categories provided in the template and do not add additional subheadings to the table. Reagents/tools that do not fit in any of the specific categories can be listed under "Other":
https://www.embopress.org/pb%2Dassets/embo-site/msb_177951_sample_FINAL.pdf

In addition, I would need from you:
- a short, two-sentence summary of the manuscript (not more than 35 words).
- three to four short bullet points highlighting the key findings of your study.
- a schematic summary figure (synopsis image) in jpeg or tiff format with the exact width of 550 pixels and a height of not more than 400 pixels that can be used as a visual synopsis on our website.

I look forward to seeing a revised version of your manuscript when it is ready. Please let me know if you have questions or comments regarding the revision.

Please use this link to submit your revision: https://embor.msubmit.net/cgi-bin/main.plex

All the best,

Achim Breiling
Senior Editor
EMBO Reports

Referee #1:

The authors have addressed all my suggestions in a very informative way. I have no further comments.

Referee #2:

I believe that this extensively revised version of the manuscript is much improved and suitable for publication in EMBO Reports

----
Referee #3:

In this revised version, authors have responded sufficiently to all my concerns. The new data with virus infection with or without Irf1 in EpiLC is a novel finding and interesting. It is unclear why the cells have to be prepared for the virus infection for such a very short period upon implantation however, this is beyond the scope of current study. With this new data, current version of the manuscript attracts more broader audience than stem cell society so it is suitable to be published in EMBO reports.

I found Fig. S7D is not properly called in the main text. I suppose this is the figure using higher concentration of virus but no information in the legend.

Response to the Reviewers

We thank all reviewers for their positive assessment of our study. We have included now a reference to the supplemental figure in the text.

1st Revision - Editorial Decision 23rd Jun 2022

Christa Buecker
Max Perutz Labs, University Vienna
Austria

Dear Dr. Buecker,

I am very pleased to accept your manuscript for publication in the next available issue of EMBO reports. Thank you for your contribution to our journal.

At the end of this email I include important information about how to proceed. Please ensure that you take the time to read the information and complete and return the necessary forms to allow us to publish your manuscript as quickly as possible.

As part of the EMBO publication's Transparent Editorial Process, EMBO reports publishes online a Review Process File to accompany accepted manuscripts. As you are aware, this File will be published in conjunction with your paper and will include the referee reports, your point-by-point response and all pertinent correspondence relating to the manuscript.

If you do NOT want this File to be published, please inform the editorial office within 2 days, if you have not done so already, otherwise the File will be published by default [contact: emboreports@embo.org]. If you do opt out, the Review Process File link will point to the following statement: "No Review Process File is available with this article, as the authors have chosen not to make the review process public in this case."

Should you be planning a Press Release on your article, please get in contact with emboreports@wiley.com as early as possible, in order to coordinate publication and release dates.

Thank you again for your contribution to EMBO reports and congratulations on a successful publication. Please consider us again in the future for your most exciting work.

Yours sincerely,

Achim Breiling
Senior Editor
EMBO Reports

****

THINGS TO DO NOW:

You will receive proofs by e-mail approximately 2-3 weeks after all relevant files have been sent to our Production Office; you should return your corrections within 2 days of receiving the proofs.

Please inform us if there is likely to be any difficulty in reaching you at the above address at that time. Failure to meet our deadlines may result in a delay of publication, or publication without your corrections.

All further communications concerning your paper should quote reference number EMBOR-2022-55375V2 and be addressed to emboreports@wiley.com.

Should you be planning a Press Release on your article, please get in contact with emboreports@wiley.com as early as possible, in order to coordinate publication and release dates.

EMBO Press Author Checklist

Corresponding Author Name: Christa Buecker	
Journal Submitted to: EMBO reports	
Manuscript Number: EMBOR-2022-55375V1	

USEFUL LINKS FOR COMPLETING THIS FORM
The EMBO Journal - Author Guidelines
EMBO Reports - Author Guidelines
Molecular Systems Biology - Author Guidelines
EMBO Molecular Medicine - Author Guidelines

Reporting Checklist for Life Science Articles (updated January 2022)
This checklist is adapted from Materials Design Analysis Reporting (MDAR) Checklist for Authors. MDAR establishes a minimum set of requirements in transparent reporting in the life sciences (see Statement of Task: 10.31222/osf.io/9sm4x). Please follow the journal's guidelines in preparing your manuscript.
Please note that a copy of this checklist will be published alongside your article.

Abridged guidelines for figures
1. Data
The data shown in figures should satisfy the following conditions:
- the data were obtained and processed according to the field's best practice and are presented to reflect the results of the experiments in an accurate and unbiased manner.
- ideally, figure panels should include only measurements that are directly comparable to each other and obtained with the same assay.
- plots include clearly labeled error bars for independent experiments and sample sizes. Unless justified, error bars should not be shown for technical replicates.
- if n<5, the individual data points from each experiment should be plotted. Any statistical test employed should be justified.
- Source Data should be included to report the data underlying figures according to the guidelines set out in the authorship guidelines on Data Presentation.

2. Captions
Each figure caption should contain the following information, for each panel where they are relevant:
- a specification of the experimental system investigated (eg cell line, species name).
- the assay(s) and method(s) used to carry out the reported observations and measurements.
- an explicit mention of the biological and chemical entity(ies) that are being measured.
- an explicit mention of the biological and chemical entity(ies) that are altered/varied/perturbed in a controlled manner.
- the exact sample size (n) for each experimental group/condition, given as a number, not a range;
- a description of the sample collection allowing the reader to understand whether the samples represent technical or biological replicates (including how many animals, litters, cultures, etc.).
- a statement of how many times the experiment shown was independently replicated in the laboratory.
- definitions of statistical methods and measures:
  - common tests, such as t-test (please specify whether paired vs. unpaired), simple χ2 tests, Wilcoxon and Mann-Whitney tests, can be unambiguously identified by name only, but more complex techniques should be described in the methods section;
  - are tests one-sided or two-sided?
  - are there adjustments for multiple comparisons?
  - exact statistical test results, e.g., P values = x but not P values < x;
  - definition of 'center values' as median or average;
  - definition of error bars as s.d. or s.e.m.

Please complete ALL of the questions below.
Select "Not Applicable" only when the requested information is not relevant for your study.

Materials

Newly Created Materials	Information included in the manuscript?	In which section is the information available? (Reagents and Tools Table, Materials and Methods, Figures, Data Availability Section)
New materials and reagents need to be available; do any restrictions apply?	Not Applicable	

Antibodies	Information included in the manuscript?	In which section is the information available? (Reagents and Tools Table, Materials and Methods, Figures, Data Availability Section)
For **antibodies** provide the following information:<br>- Commercial antibodies: RRID (if possible) or supplier name, catalogue number and or/clone number<br>- Non-commercial: RRID or citation	Yes	Reagent and Tools Table

DNA and RNA sequences	Information included in the manuscript?	In which section is the information available? (Reagents and Tools Table, Materials and Methods, Figures, Data Availability Section)
**Short novel DNA or RNA including primers, probes**: provide the sequences.	Yes	Reagent and Tools Table

Cell materials	Information included in the manuscript?	In which section is the information available? (Reagents and Tools Table, Materials and Methods, Figures, Data Availability Section)
**Cell lines:** Provide species information, strain. Provide accession number in repository **OR** supplier name, catalog number, clone number, and/**OR** RRID.	Yes	Material and Methods
**Primary cultures:** Provide species, strain, sex of origin, genetic modification status.	Not Applicable	
Report if the cell lines were recently **authenticated** (e.g., by STR profiling) and tested for mycoplasma contamination.	Yes	Material and Methods. Cell lines were tested regularly for mycoplasma contamination

Experimental animals	Information included in the manuscript?	In which section is the information available? (Reagents and Tools Table, Materials and Methods, Figures, Data Availability Section)
**Laboratory animals or Model organisms:** Provide species, strain, sex, age, genetic modification status. Provide accession number in repository **OR** supplier name, catalog number, clone number, **OR** RRID.	Not Applicable	
**Animal observed in or captured from the field:** Provide species, sex, and age where possible.	Not Applicable	
Please detail **housing and husbandry conditions**.	Not Applicable	

Plants and microbes	Information included in the manuscript?	In which section is the information available? (Reagents and Tools Table, Materials and Methods, Figures, Data Availability Section)
**Plants:** provide species and strain, ecotype and cultivar where relevant, unique accession number if available, and source (including location for collected wild specimens).	Not Applicable	
**Microbes:** provide species and strain, unique accession number if available, and source.	Not Applicable	

Human research participants	Information included in the manuscript?	In which section is the information available? (Reagents and Tools Table, Materials and Methods, Figures, Data Availability Section)
If collected and within the bounds of privacy constraints report on age, sex and gender or ethnicity for all study participants.	Not Applicable	

Core facilities	Information included in the manuscript?	In which section is the information available? (Reagents and Tools Table, Materials and Methods, Figures, Data Availability Section)
If your work benefited from core facilities, was their service mentioned in the acknowledgments section?	Yes	Acknowledgments

Design

Study protocol	Information included in the manuscript?	In which section is the information available? (Reagents and Tools Table, Materials and Methods, Figures, Data Availability Section)
If study protocol has been **pre-registered, provide DOI in the manuscript**. For clinical trials, provide the trial registration number **OR** cite DOI.	Not Applicable	
Report the **clinical trial registration number** (at ClinicalTrials.gov or equivalent), where applicable.	Not Applicable	

Laboratory protocol	Information included in the manuscript?	In which section is the information available? (Reagents and Tools Table, Materials and Methods, Figures, Data Availability Section)
Provide DOI OR other citation details if **external detailed step-by-step protocols** are available.	Not Applicable	

Experimental study design and statistics	Information included in the manuscript?	In which section is the information available? (Reagents and Tools Table, Materials and Methods, Figures, Data Availability Section)
Include a statement about **sample size** estimate even if no statistical methods were used.	Yes	Material and Methods
Were any steps taken to minimize the effects of subjective bias when allocating animals/samples to treatment (e.g. **randomization procedure**)? If yes, have they been described?	Not Applicable	
Include a statement about **blinding** even if no blinding was done.	Not Applicable	There was no blinding done
Describe **inclusion/exclusion criteria** if samples or animals were excluded from the analysis. Were the criteria pre-established?<br><br>If sample or data points were omitted from analysis, report if this was due to attrition or intentional exclusion and provide justification.	Not Applicable	
For every figure, are **statistical tests** justified as appropriate? Do the data meet the assumptions of the tests (e.g., normal distribution)? Describe any methods used to assess it. Is there an estimate of variation within each group of data? Is the variance similar between the groups that are being statistically compared?	Yes	Figures

Sample definition and in-laboratory replication	Information included in the manuscript?	In which section is the information available? (Reagents and Tools Table, Materials and Methods, Figures, Data Availability Section)
In the figure legends: state number of times the experiment was **replicated** in laboratory.	Yes	Figures
In the figure legends: define whether data describe **technical or biological replicates**.	Yes	Figures

Ethics

Ethics	Information included in the manuscript?	In which section is the information available? (Reagents and Tools Table, Materials and Methods, Figures, Data Availability Section)
Studies involving **human participants**: State details of **authority granting ethics approval** (IRB or equivalent committee(s), provide reference number for approval.	Not Applicable	
Studies involving **human participants**: Include a statement confirming that **informed consent** was obtained from all subjects and that the experiments conformed to the principles set out in the WMA Declaration of Helsinki and the Department of Health and Human Services Belmont Report.	Not Applicable	
Studies involving **human participants:** For publication of **patient photos**, include a statement confirming that consent to publish was obtained.	Not Applicable	
Studies involving experimental **animals**: State details of **authority granting ethics approva**l (IRB or equivalent committee(s), provide reference number for approval. Include a statement of compliance with ethical regulations.	Not Applicable	
Studies involving **specimen and field samples:** State if relevant **permits** obtained, provide details of authority approving study; if none were required, explain why.	Not Applicable	

Dual Use Research of Concern (DURC)	Information included in the manuscript?	In which section is the information available? (Reagents and Tools Table, Materials and Methods, Figures, Data Availability Section)
Could your study fall under dual use research restrictions? Please check biosecurity documents and list of **select agents and toxins** (CDC): https://www.selectagents.gov/sat/list.htm	Not Applicable	
If you used a select agent, is the security level of the lab appropriate and reported in the manuscript?	Not Applicable	
If a study is subject to dual use research of concern regulations, is the name of the **authority granting approval and reference number** for the regulatory approval provided in the manuscript?	Not Applicable	

Reporting

The MDAR framework recommends adoption of discipline-specific guidelines, established and endorsed through community initiatives. Journals have their own policy about requiring specific guidelines and recommendations to complement MDAR.

Adherence to community standards	Information included in the manuscript?	In which section is the information available? (Reagents and Tools Table, Materials and Methods, Figures, Data Availability Section)
State if relevant guidelines or checklists (e.g., **ICMJE, MIBBI, ARRIVE, PRISMA**) have been followed or provided.	Not Applicable	
For **tumor marker prognostic studies,** we recommend that you follow the **REMARK** reporting guidelines (see link list at top right). See author guidelines, under 'Reporting Guidelines'. Please confirm you have followed these guidelines.	Not Applicable	
For **phase II and III randomized controlled trials**, please refer to the **CONSORT** flow diagram (see link list at top right) and submit the CONSORT checklist (see link list at top right) with your submission. See author guidelines, under 'Reporting Guidelines'. Please confirm you have submitted this list.	Not Applicable	

Data Availability

Data availability	Information included in the manuscript?	In which section is the information available? (Reagents and Tools Table, Materials and Methods, Figures, Data Availability Section)
Have **primary datasets** been deposited according to the journal's guidelines (see 'Data Deposition' section) and the respective accession numbers provided in the Data Availability Section?	Yes	Data Availability Section
Were **human clinical and genomic datasets** deposited in a public access-controlled repository in accordance to ethical obligations to the patients and to the applicable consent agreement?	Not Applicable	
Are **computational models** that are central and integral to a study available without restrictions in a machine-readable form? Were the relevant accession numbers or links provided?	Not Applicable	
If publicly available data were reused, provide the respective **data citations in the reference list**.	Yes	Data Citation Format
